# Purification and initial characterization of *Plasmodium falciparum* K^+^ channels, PfKch1 and PfKch2 produced in *Saccharomyces cerevisiae*

**DOI:** 10.1186/s12934-020-01437-7

**Published:** 2020-09-21

**Authors:** Karen Molbaek, Maria Tejada, Christina Hoeier Ricke, Peter Scharff-Poulsen, Peter Ellekvist, Claus Helix-Nielsen, Nirbhay Kumar, Dan A. Klaerke, Per Amstrup Pedersen

**Affiliations:** 1 Department of Veterinary and Animal Sciences, University of Copenhagen, Frederiksberg, 1870 Denmark; 2 Department of Biology, University of Copenhagen, Copenhagen, 2100 Denmark; 3 Medical Department, Herlev-Gentofte Hospital, Herlev, 2730 Denmark; 4 Aquaporin A/S, Kgs Lyngby, 2800 Denmark; 5 Department of Environmental Engineering, Technical University of Denmark, Kgs Lyngby, 2800 Denmark; 6 University of Maribor, Laboratory for Water Biophysics and Membrane Technology, Maribor, 2000 Slovenia; 7 Department of Global Health, Milken Institute School of Public Health, George Washington University, Washington DC, 20052-0066 USA

**Keywords:** Malaria, K-channels, Yeast, Recombinant protein

## Abstract

Resistance towards known antimalarial drugs poses a significant problem, urging for novel drugs that target vital proteins in the malaria parasite *Plasmodium falciparum*. However, recombinant production of malaria proteins is notoriously difficult. To address this, we have investigated two putative K^+^ channels, PfKch1 and PfKch2, identified in the *P. falciparum* genome. We show that PfKch1 and PfKch2 and a C-terminally truncated version of PfKch1 (PfKch1^1−1094^) could indeed be functionally expressed in vivo, since a K^+^-uptake deficient *Saccharomyces cerevisiae* strain was complemented by the *P. falciparum* cDNAs. PfKch1^1−1094^-GFP and GFP-PfKch2 fusion proteins were overexpressed in yeast, purified and reconstituted in lipid bilayers to determine their electrophysiological activity. Single channel conductance amounted to 16 ± 1 pS for PfKch1^1−1094^-GFP and 28 ± 2 pS for GFP-PfKch2. We predicted regulator of K^+^-conductance (RCK) domains in the C-terminals of both channels, and we accordingly measured channel activity in the presence of Ca^2+.^

## Introduction

The recent decade has experienced a dramatic decrease in malaria prevalence and morbidity, which is partly due to combined efforts including rapid diagnostics, prompt treatment based on artemisinin combination therapies and the use of insecticide treated nets [1]. However, an estimated 228 million cases still occur annually, leading to around 405,000 deaths a year [2], and the occurrence of resistance towards several artemisinin based combination therapies now calls for the development of new drugs [3, 4], preferably aimed at new targets. A recent reverse genetic screen of the murine malaria model *P. berghei,* using the PlasmoGEM database knock out vector library [5], suggested that a staggering two-thirds of its genome may be essential for normal intra-erythrocytic growth [6].

Bioinformatics analysis of the *P. falciparum* genome has revealed new potential drug targets such as membrane transporters, channels and pores [7, 8]. A number of membrane transporters from *P. falciparum* has subsequently been cloned and characterized in *Xenopus laevis* oocytes [9–13] and some have been shown to be crucial to asexual parasite development. Two genes encoding putative K^+^ channels PfKch1(Uniprot Q8I5E6) and PfKch2 (Uniprot Q8IKI3) were identified in the *P. falciparum* genome by searching for the amino acid sequence of the highly conserved pore-loop domain [7, 14, 15]. Attempts to establish PfKch1 and PfKch2 knock outs were unsuccessful leading to the suggestion that they may be critical for asexual parasite development [15]. PbKch1, the murine homologue of PfKch1, mediates K^+^ transport in *P. berghei* parasites and, in addition, a high K^+^ concentration has been shown to promote sporozoite infectivity in cell cultures [16]. Gene disruption studies with PbKch1 showed that this channel in *P. berghei* is essential for sexual-stage parasite development in the mosquito vector [17]. PbKch2, however, has recently been examined in a genetic knock out model and found to be non-essential for K^+^ transport across the parasite plasma membrane, whether in the intra-erythrocytic or in the mosquito residing stages [18]. Thus, the specific function of PbKch2 function remains unknown. In general, K^+^ channels have are excellent targets in drug development, and the potential for the *Plasmodium* channels as targets for treatment of malaria has been discussed in several reviews (see e.g., 7, 8, 19).

**Fig. 1:**
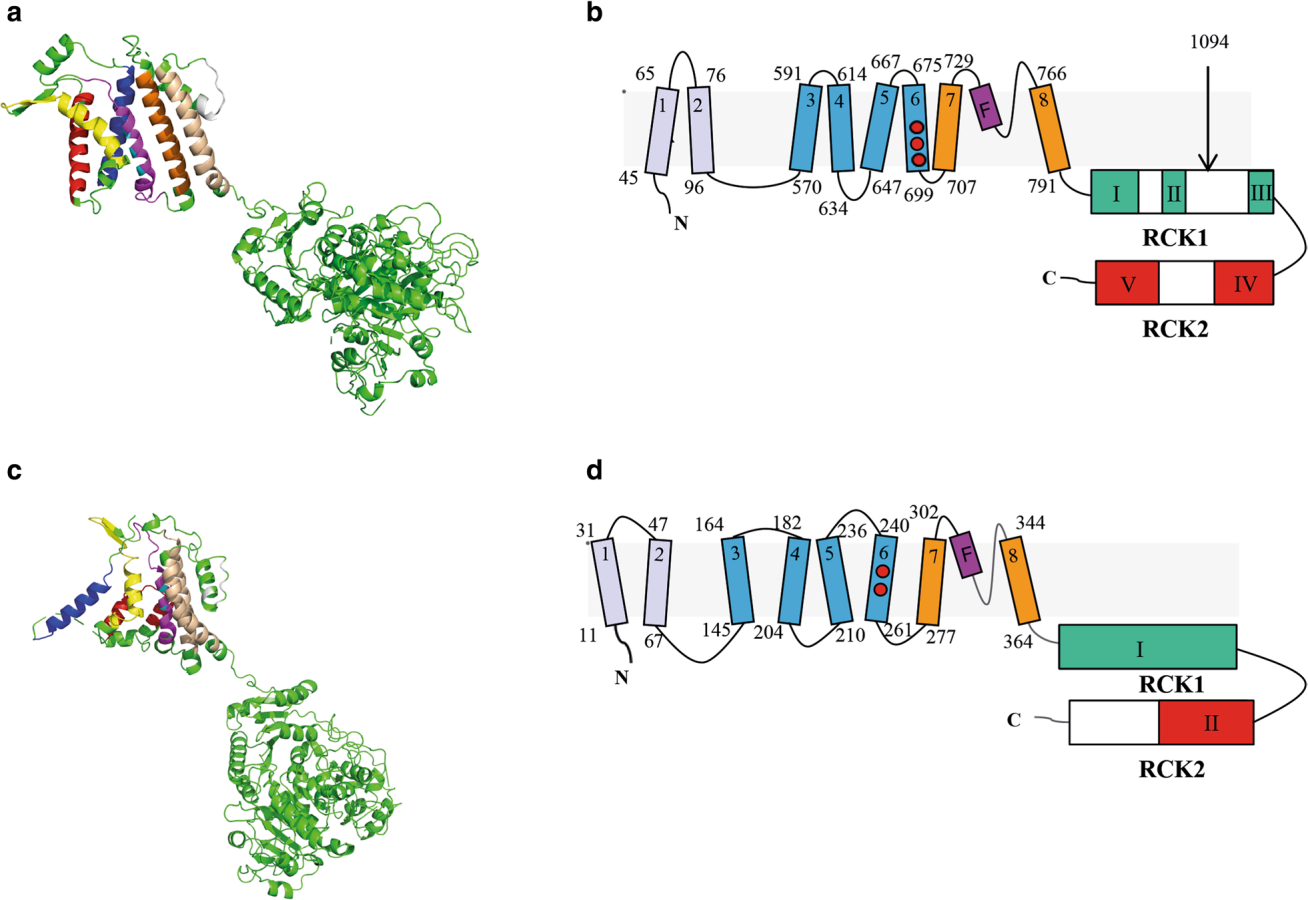
Modelling and membrane topology prediction of PfKch1^fl^ and PfKch2^fl^. Homology modelling of residues 570 —1966 of PfKch1 (**a**) and residues 140—1461 of PfKch2 (**c**) using Phyre^2^ and the transmembrane topologies of full length PfKch1 (**b**) and full length PfKch2 (**d**) using a combination of Phyre^2^, TOPCONS and TMHMM (**b, d**) (see Additional file 1: Fig.S1). The Phyre^2^ modelled transmembrane helixes TM3 to TM8 are colored blue, red, yellow, purple, orange and tints, respectively. The positions of Arginine and Lysine residues potentially involved in voltage dependency are shown in cyan while the consensus pore region is depicted in gray. Numbers in the topology models indicate amino acids initiating or termination transmembrane helixes. Red spheres show the location of positively charged amino acids presumably involved in voltage dependent gating. Helices involved in possible voltage sensing are shown in blue while helices involved in K^+^ selectivity are depicted in orange. The pore signature sequence TXXTXGYG is shown in purple. The locations of predicted RCK1 and RCK 2 domains (regulator of conductance of K + channels) are shown in green and red, respectively, and numbered I, II, III, IV and V. White boxes indicate insertions that are not found in the SLO1 channels from e.g. man and zebrafish. An arrow indicates the position of the C-terminal end of the truncated PfKch1^1−1094^ channel

K^+^ channels are large and complex integral membrane proteins, assembling as either homo-dimers or homo-tetramers. Despite their widespread abundance, importance for all living organisms and potential as drug targets [20, 21], high resolution structures are not available for eukaryotic parasite K^+^ channels [22]. The limited structural information is not a result of lack of interest, but reflects the huge difficulties associated with expressing and purifying biologically active membrane proteins in general and K^+^ channels in particular. On top of this recombinant production of *P. falciparum* proteins is notoriously difficult [23, 24]. This is certainly true for the PfKch1 and PfKch2 channels, which are large proteins of 1,966 and 1,461 amino acids, respectively, that presumably have to assemble into homo- tetramers to become functional. The two channels furthermore have extraordinary long C-terminal regions with unusual primary structures (Fig. 1).

Due to the limited knowledge of the *P. falciparum* K^+^ channels the aim of the present paper was to develop an expression and purification protocol for two difficult to express *P. falciparum* K^+^ channels and initiate an electrophysiological characterization of the purified channel proteins. In addition to contributing to our understanding of the biology of the parasite, access to pure and active protein may allow screening for channel inhibitors with the potential of being antimalarial medicine. As the two *P. falciparum* channels have resisted expression in traditional expression systems for ion channels, such as *Xenopus* oocyte and mammalian cells, the only option seems to be characterization of the purified channel proteins.

## Results

### The *S. cerevisiae* platform for expression of *P. falciparum* K^+^ channels

Since our previous attempts to express PfKch1 and Pfkch2 in a number of hosts have been unsuccessful (*unpublished results*) we decided to explore production of PfKch1 and PfKch2 in our *S. cerevisiae* expression platform that has turned out to be efficient for high-yield and high-quality production of eukaryotic membrane proteins [25–27].

**Fig. 2:**
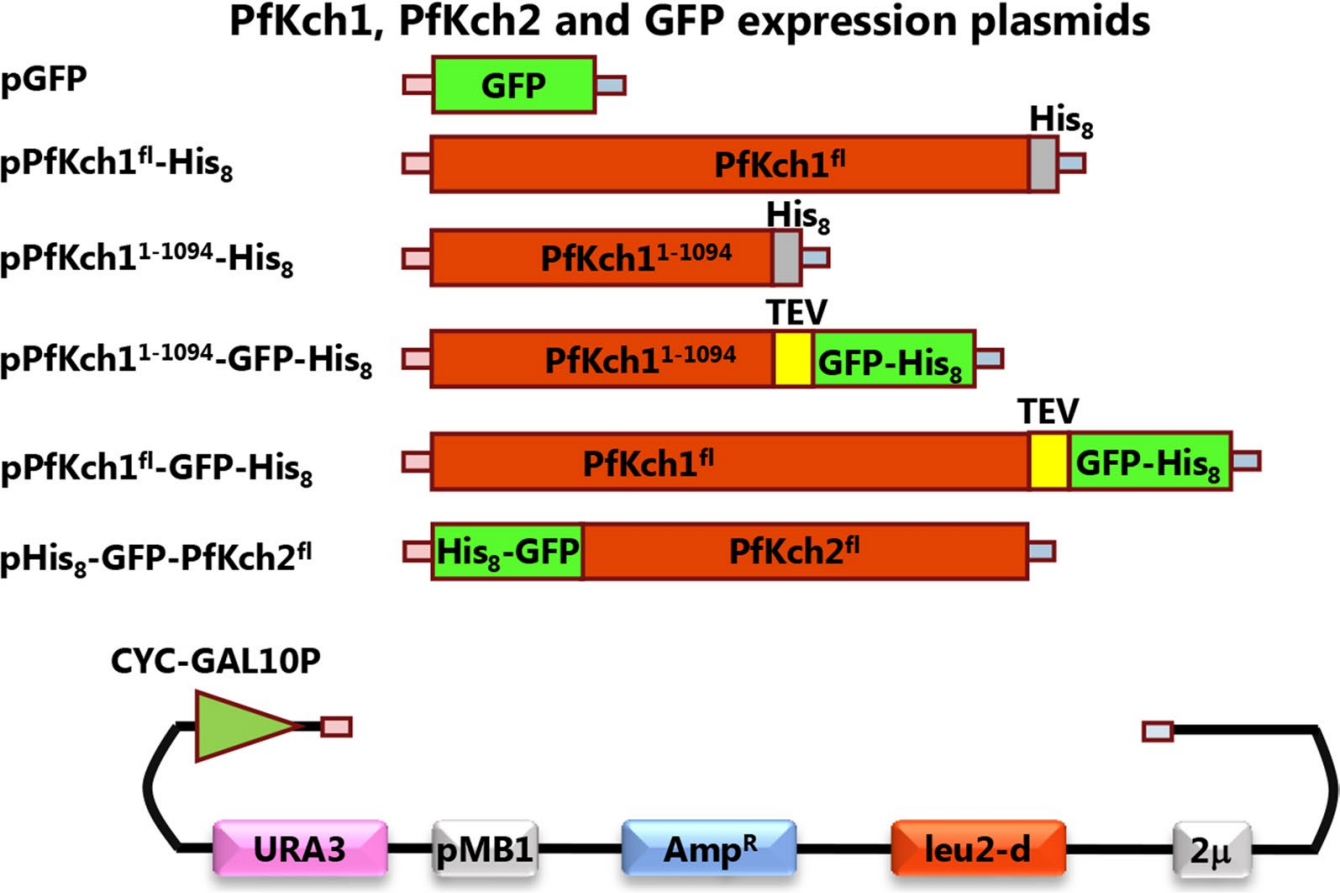
Structural map of the PfKch1-GFP and GFP-PfKch2 expression plasmids. CYC-GAL10P, a hybrid promoter carrying the non-translated leader of the cytochrome-1 gene fused to the GAL10 upstream activating sequence; TEV, a Tobacco Etch Virus cleavage site; GFP-His_8_, yeast enhanced GFP cDNA with eight subsequent histidine codons; 2µ, yeast 2 micron origin of replication; leu2-d, a poorly expressing allele of the β-isopropylmalate dehydrogenase gene; bla, a β-lactamase gene; pMB1, the pMB1 origin of replication; URA3, the yeast orotidine-5′-phosphate decarboxylase gene. Expression plasmid construction was done by insertion of either PfKch1/PfKch2 PCR fragments or PfKch1/PfKch2 and GFP PCR fragments into the linearized expression vector pEMBLyex4 by in vivo homologous recombination in *S. cerevisiae*. Regions used for homologous recombination are shown in pink and grey respectively

For heterologous expression of the *Plasmodium* channels in yeast, plasmids as depicted in Fig. 2 were generated. Expression from these plasmids can easily be manipulated due to the galactose inducible promoter and an adjustable plasmid copy number. Selection for uracil autotrophy results in a plasmid copy number of approximately 20, while selection for leucine autotrophy further increases the number of plasmids to approximately 200 copies per cell [28]. To benefit from the ultrahigh plasmid copy number we have engineered a yeast strain to over express the Gal4 transcriptional activator in a regulated way. Gal4 is required for induction of the galactose inducible promoter located on the expression plasmids [29]. The GFP tag was placed N-terminally in the Pfkch2 construct, as it did not express well when the tag was placed C-terminally.

### GFP tagged PfKch1^1−1094^, PfKch1^fl^ and PfKch2^fl^ mediate K^+^ uptake in *S. cerevisiae*

**Fig. 3:**
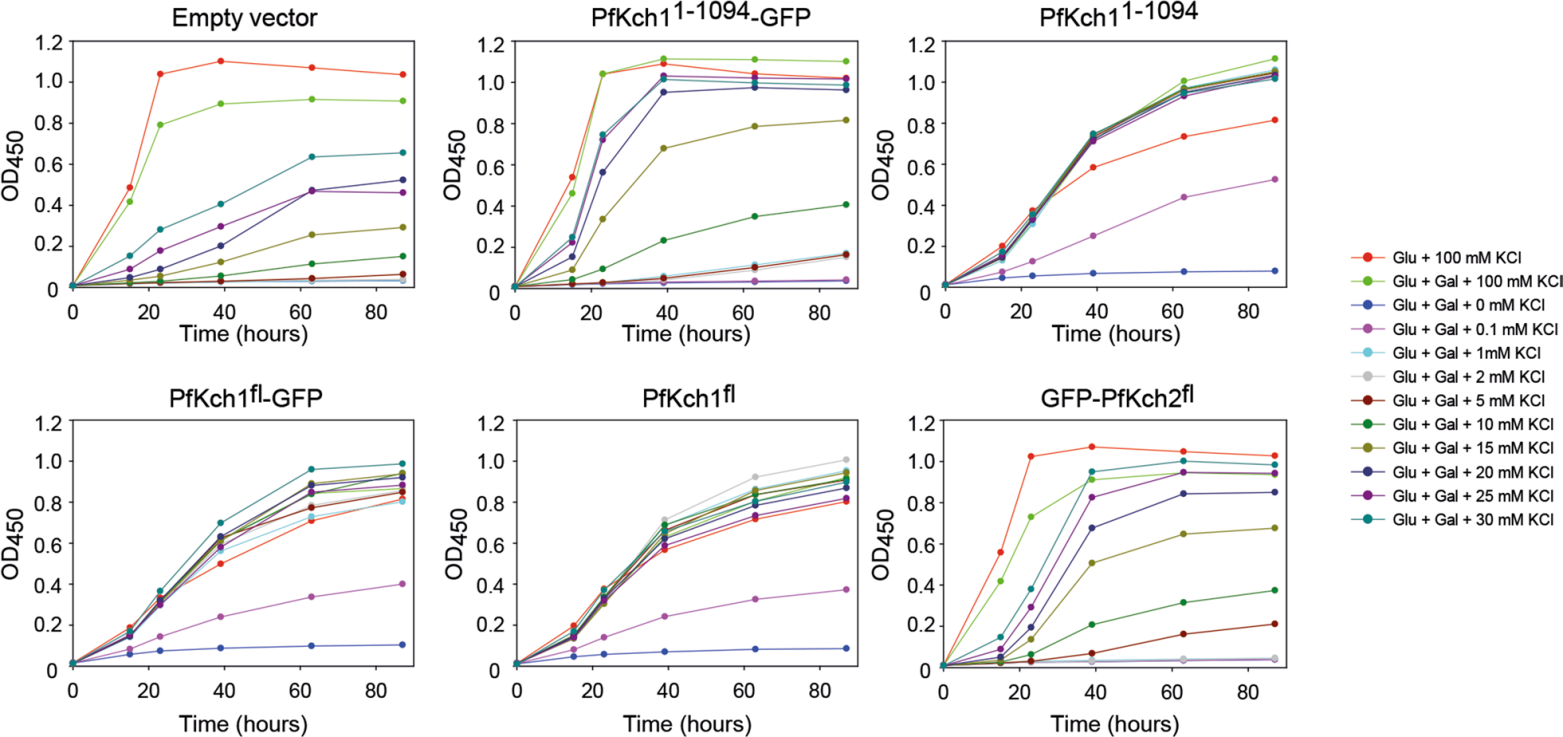
The *P. faliciparum* K-channels complement the high KCl requirement of a *trk1*Δ, *trk2*Δ yeast strain Cells growing exponentially in glucose minimal medium supplemented with 100 mM KCl were harvested by centrifugation, washed four times in 18 mΩ water, re-suspended to OD_450_ = 0.5 in 18 mΩ water and used for inoculation of growth media in microplates containing different concentrations of KCl as indicated in the figure and described in the Methods section. OD_450_ in each well was measured twice a day. All experiments were performed in triplicate. Only a GFP tagged version of PfKch2^fl^ was included in this study

Before initiating large scale production and development of purification protocols, we wished to establish whether *S. cerevisiae* can produce and assemble the complex *P. falciparum* channels in a functional form in the plasma membrane. For this purpose we employed the *S. cerevisiae* strain CY162 [30], which is devoid of high affinity K^+^ uptake, and therefore can be used to assay for in vivo activity of the heterologous channels. We analyzed the ability of GFP-tagged and non-tagged versions of PfKch1^1−1094^ and PfKch1^fl^ channels (see Fig. 2) to complement the requirement for a high extra cellular K^+^ concentration for growth of the CY162 yeast strain. As the C-terminal of PfKchn1 is particularly long and has a peculiar amino acid composition, we decided to include a truncated version lacking the 885 C-terminal amino acids, PfKchn1^1−1094^. To get detailed information on the complementation capacity of the PfKch1 and PfKch2 constructs, we used a micro plate-based growth assay using 11 different KCl concentrations. As seen from Fig. 3 the CY162 strain carrying the empty expression plasmid grew perfectly well at a KCl concentration of 100 mM. However, growth was severely affected at 30 mM KCl and further decreased as the KCl concentration was reduced to 10 mM. Growth ceased at KCl concentrations lower than 10 mM. For CY162 cells carrying either the PfKch1^1−1094^ or the PfKch1^fl^ expression plasmid, growth was unaffected until the KCl concentration was reduced to 0.1 mM (Fig. 3). As expected, absence of KCl in the growth medium was prohibitive for cell proliferation. It can also be seen from Fig. 3 that the ability of PfKch1^1−1094^-GFP to complement the growth defect of CY162 cells required a higher external KCl concentration than the non-tagged channel. GFP tagging of the truncated channel was found to impair growth more severely than tagging of the full length channel, as comparison of the two upper right panels (PfKch1^1−1094^ and PfKch1^1−1094^-GFP) shows a bigger difference in growth at any given KCl concentration, than the two lower left panels (PfKch1^fl^ and PfKch1^fl^-GFP).

Comparing the KCl dependent growth profiles of GFP tagged PfKch2^fl^ and empty vector revealed that PfKch2^fl^ conferred weaker complementation than PfKch1^fl^ or PfKch1^1−1094^, but growth was still improved compared to that of cells carrying the empty expression vector. After 40 h the OD_450_ of Pfkch2^fl^ cultures grown in 15 mM KCl or more, all exceeded 0.5, whereas the corresponding cultures for the empty vector control cultures were all below 0.5.

**Fig. 4:**
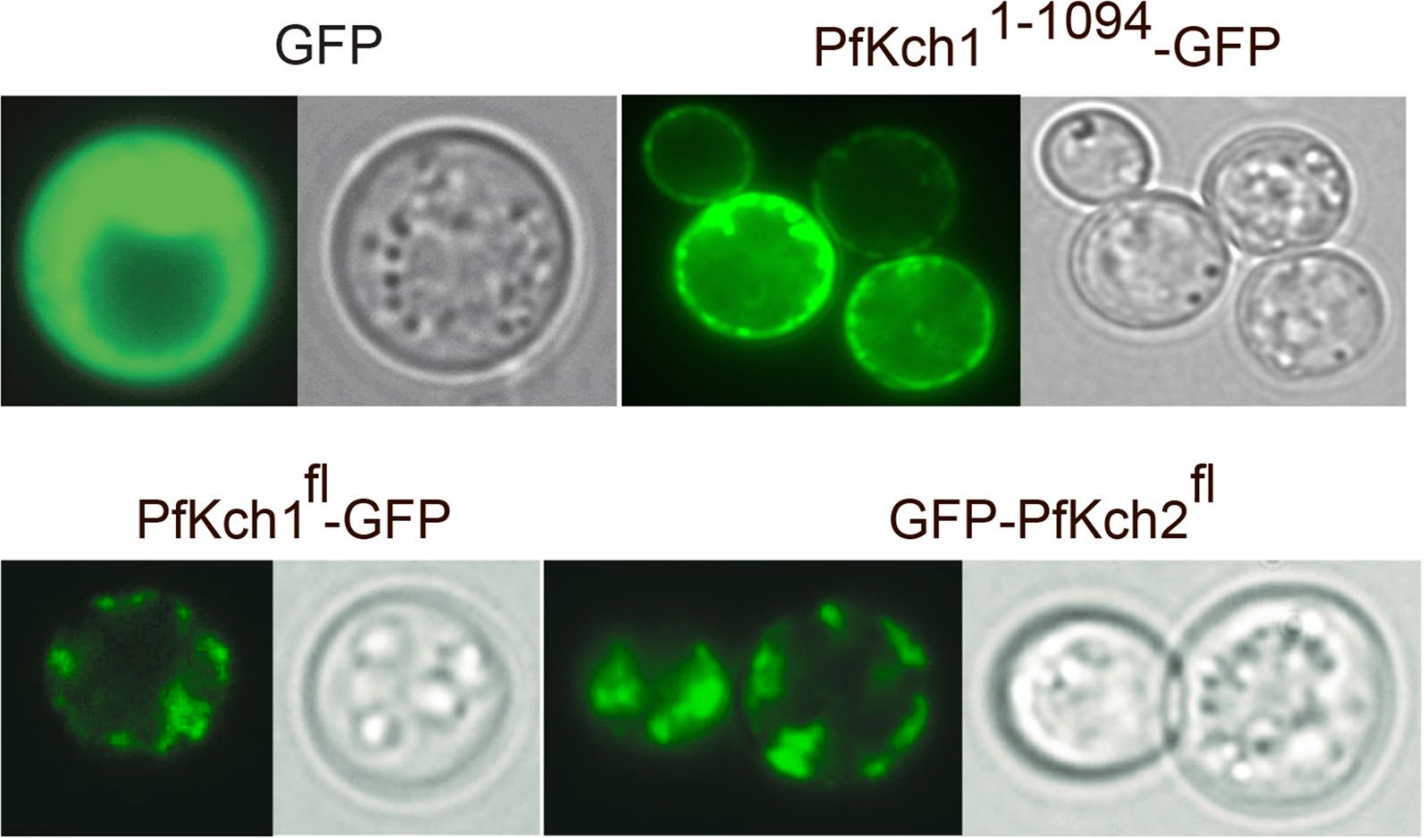
Bioimaging of CY162 producing GFP, PfKch1^fl^-GFP, PfKch1^1−1094^-GFP or GFP-PfKch2^fl^. Yeast cells were grown in expression medium supplemented with 100 mM KCl at room temperature until OD_450_ = 1.0, transferred to 15 °C and induced with 2% galactose media for 24 h. Left panels, GFP fluorescence; right panels, differential interference contrast image (DIC)

To determine if the observed differences in complementation capacity of PfKch1^fl^, PfKch1^1−1094^ and PfKch2^fl^ reflected their localization in yeast we used bioimaging of the GFP tagged proteins to observe whether they localize to the plasma membrane or intracellular membranes. It can be seen from Fig. 4 that while GFP tagged PfKch1^1−1094^ mainly localized to the plasma membrane, PfKch1^fl^-GFP showed a less distinct localization since GFP fluorescence was observed in the plasma membrane as well as in intracellular structures. GFP-PfKch2^fl^ was almost entirely localized to intracellular membranes. The reduced complementation capacity of GFP-PfKch2^fl^ may therefore result from its accumulation primarily in intracellular membranes. As expected, fluorescence was diffusely located in the cytoplasm when only GFP was expressed.

### GFP-tagged PfKch1^1−1094^, PfKch1^fl^, and PfKch2^fl^ also show different localization in the PAP1500 production strain

**Fig. 5:**
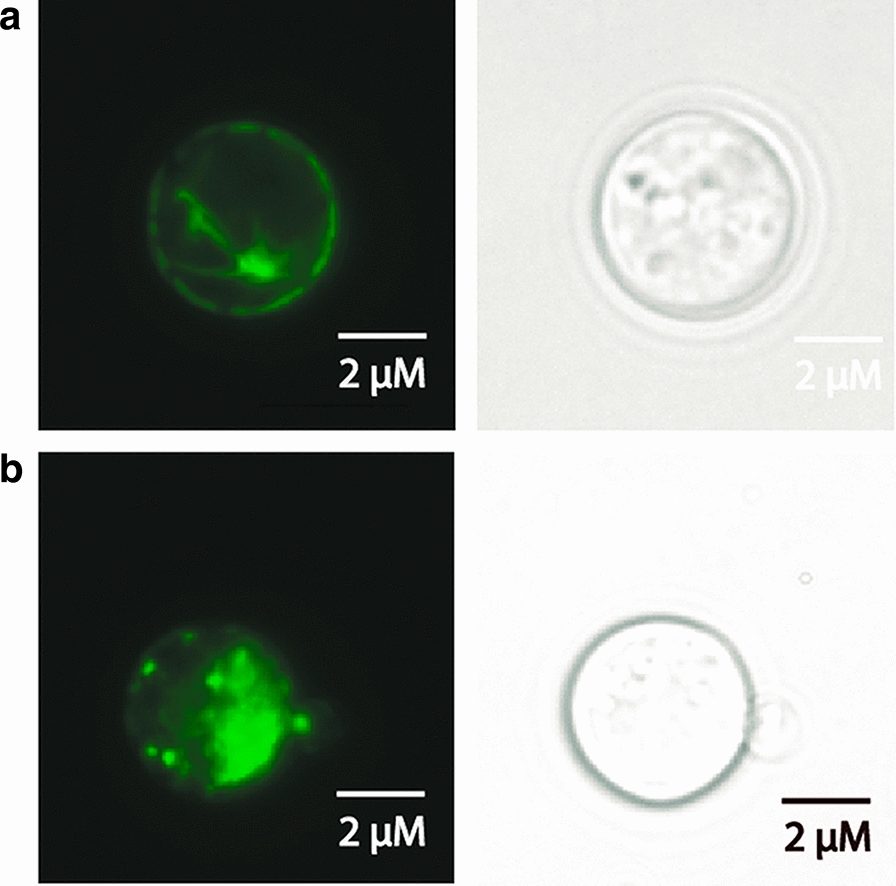
Live cell bio-imaging of GFP-tagged PfKch1^1−1094^ and PfKch2^fl^ produced in the PAP1500 production strain. Yeast cell cultures were grown at room temperature in expression medium until OD_450_ = 1.0, transferred to 15 °C and after were temperature equilibration they were induced with 2% galactose for 24 h. a: PfKch1^1−1094^-GFP; b: GFP-PfKch2^fl^. Left panels: GFP fluorescence; right panels: differential interference contrast image (DIC)

To discriminate between localization in the plasma membrane and in intracellular structures,we performed live cell bioimaging of the production strain, PAP1500, expressing the three constructs. PfKch1^fl^-GFP did not express well in this strain, and with barely detectable fluorescence levels we decided to focus on expression and purification of GFP-PfKch2^fl^ and PfKch1^1−1094^-GFP. Data in Fig. 5 show that PfKch1^1−1094^-GFP localized almost exclusively to the plasma membrane (Fig. 5a). In contrast GFP-PfKch2^fl^ accumulated primarily in internal structures (Fig. 5b). GFP fluorescence emitted from the fusion proteins indicates that they have maintained their folding in the production strain [31].

### Accumulation of recombinant PfKch1^1−1094^-GFP and GFP-PfKch2^fl^ peaks at 15 °C

**Fig. 6:**
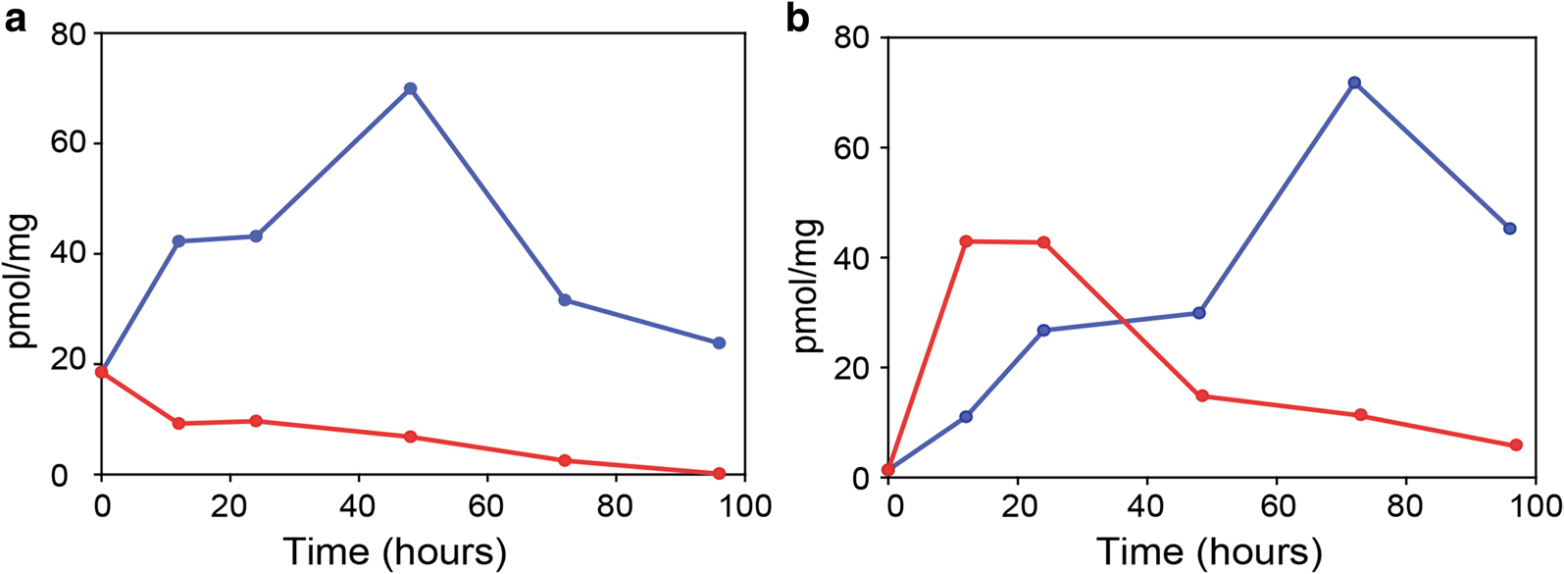
Time and temperature dependent accumulation of PfKch1^1−1094^-GFP and GFP-PfKch2^fl^. Yeast cell cultures were grown at room temperature (20-25 °C) and at OD_450_ = 1.0, they were separated in two. One was subsequently incubated at 15 °C and the other at 30 °C. After thermo-equilibration, production of GFP tagged channels was induced by addition of galactose (t = 0). Crude membranes were prepared from cells harvested at the indicated time points. GFP Fluorescence was measured in crude membranes, and translated into pmol channel protein/mg total membrane protein using a GFP standard curve. 15 °C (blue curve) or 30 °C (red curve). a: PfKch1^1−1094^-GFP. b: GFP-PfKch2^fl^

We have previously shown that a reduction in expression temperature is beneficial for recombinant membrane protein accumulation in our expression system [25–27]. The data in Fig. 6 show that this was also the case for the two *P. falciparum* K^+^ channels. PfKch1^1−1094^-GFP accumulated to approximately 70 pmol/mg crude membrane protein at 15 °C after 48 h induction corresponding to approximately 1.2% of total membrane protein content. For GFP-PfKch2^fl^ the yield after 72 h at 15 °C was 72 pmol/mg corresponding to approximately 1.5% of total membrane protein content. When expressed at 30 °C PfKch1^1−1094^-GFP did not accumulate significantly (Fig. 6a) while GFP-PfKch2^fl^ accumulation at this temperature peaked at some 40 pmol/mg total protein after 12–24 h, but then drastically declined (Fig. 6b).

### Detergent screen reveals suitable solubilization conditions

**Fig. 7:**
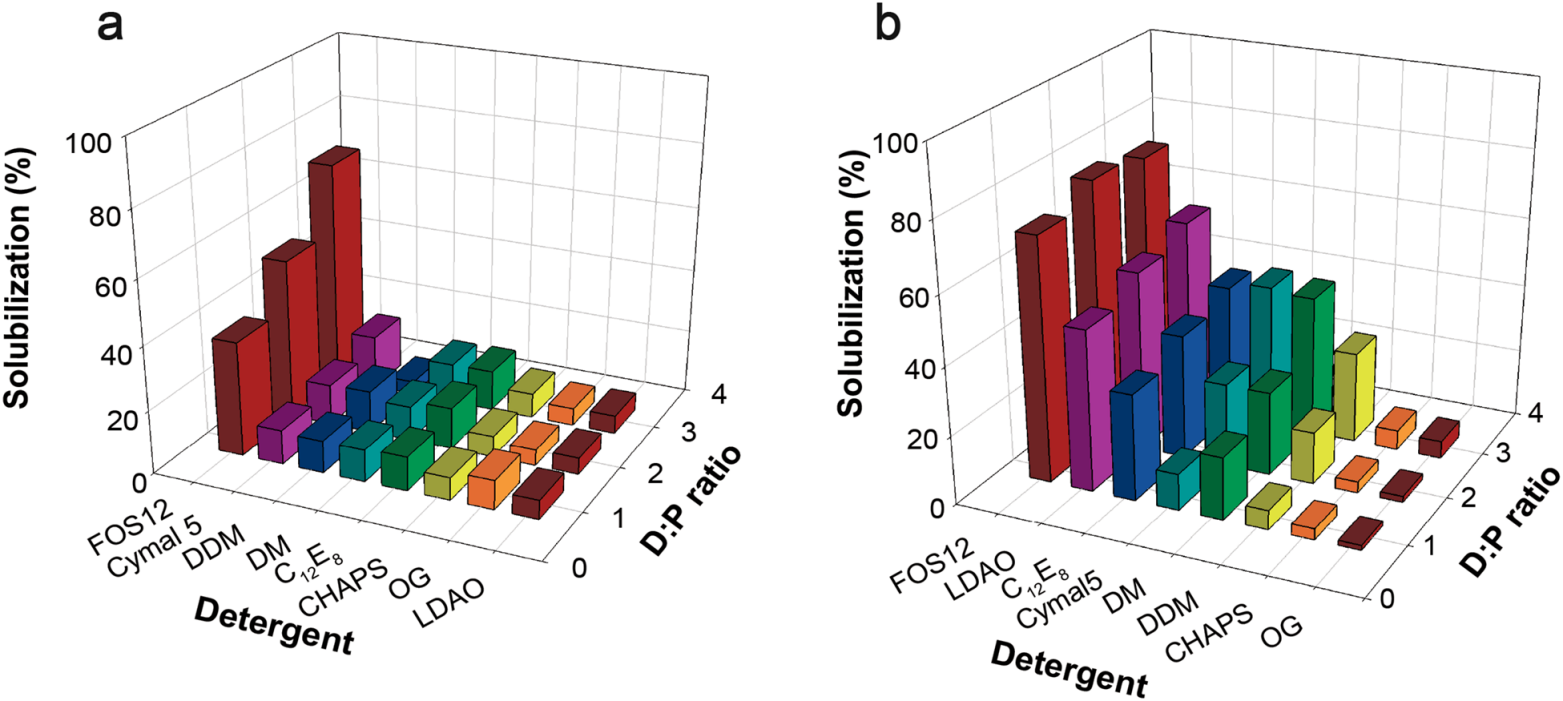
Detergent screen for solubilization of GFP tagged PfKch1^1−1094^ and PfKch2^fl^ from crude membranes. Membrane proteins PfKch1^1−1094^-GFP (**a**) and GFP-PfKch2^fl^ (**b**) were solubilized using the indicated detergent/protein ratios and a cholesteryl-hemisuccinate concentration of 0.68, 1.36 or 2 mg/ml for the 3 ratios, respectively. Abbreviations; FOS-12, Fos-Choline-12; LDAO lauryldimethylamine N-oxide; Cymal5, 5-Cyclohexyl-1-pentyl-β-d-maltoside; DDM, n-Dodecyl-β-d-maltopyranoside; DM, n-Decyl-β-d-maltopyranoside; C_12_E_8_, Octaethylene glycol monododecyl ether; CHAPS, 3-[(3-cholamidopropyl) dimethylam-monio] -1-propanesulfonate; OG, n-Octyl-β-d-glucopyranoside. Solubilization efficiency was defined as GFP fluorescence of solubilized protein normalized to GFP fluorescence in the crude membranes used for solubilization

Several detergents were screened to determine the most efficient ones for solubilization of each channel (Fig. 7). Our previous work [27] showed that addition of cholesteryl-hemisuccinate (CHS) stabilized the hERG K^+^ channel during and after extraction from the membranes. We therefore included CHS in our detergent screen. Figure 7 shows that PfKch1^1−1094^-GFP (a) and GFP-PfKch2^fl^ (b) were most efficiently solubilized from crude yeast membranes at protein:FOS-12:CHS ratios of 1:3:1.5 (w/w/w), giving solubilization yields of more than 70% for each. GFP-PfKch2^fl^ could also be solubilized at yields of at least 50% in LDAO, and in the vicinity of 40% using DM, Cymal-5 or C_12_E_18_ (Fig. 7b).

### Cholesterol improves monodispersity of detergent solubilized channel proteins

**Fig. 8:**
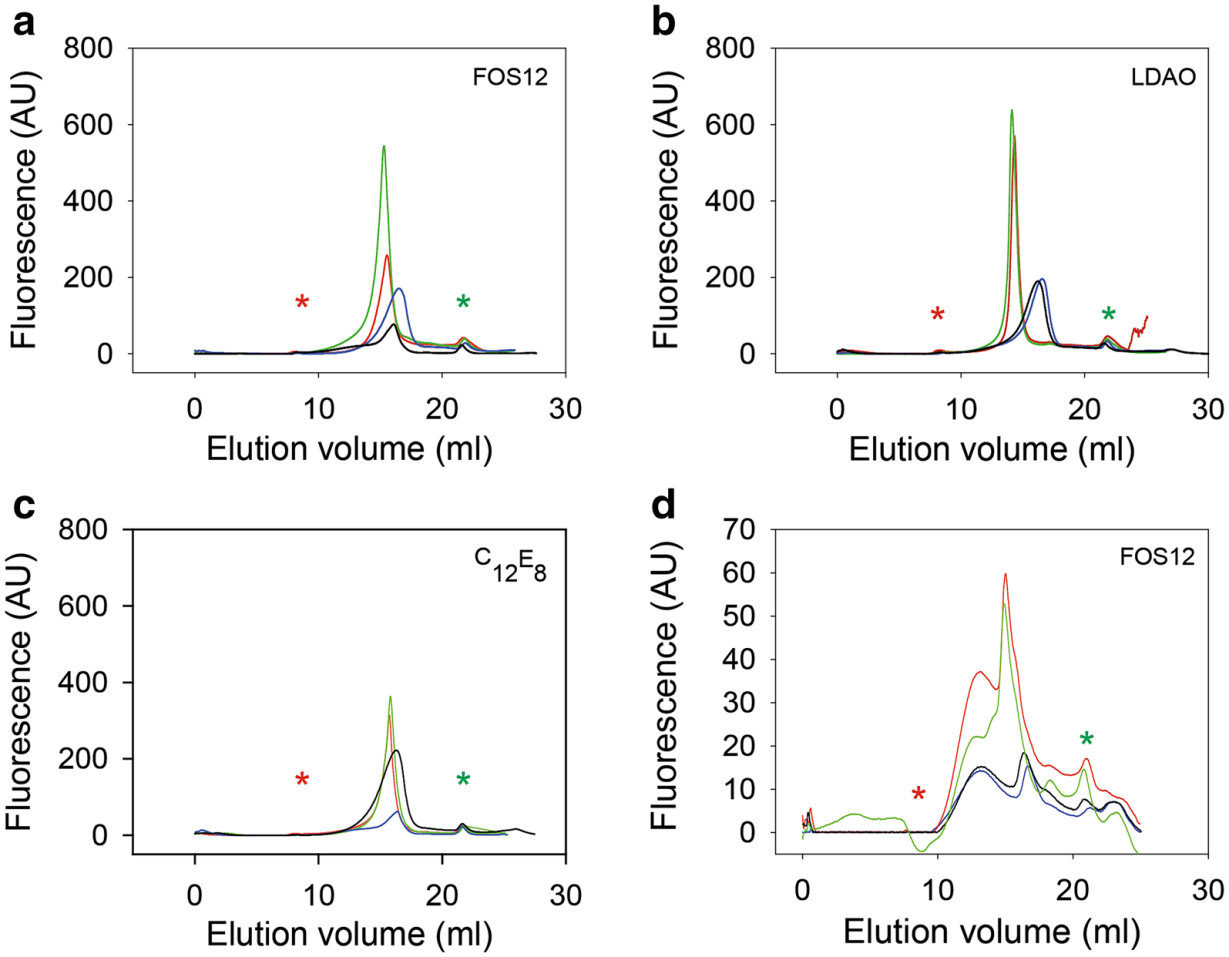
FSEC profiles of detergent solubilized PfKch1^1−1094^-GFP and GFP-PfKch2^fl^. GFP-PfKch2^fl^ in FOS-12 (**a**), LDAO (**b**), C_12_E_8_ (C) and PfKch1^1−1094^-GFP in FOS-12 (**d**) at 3:1 detergent to protein ratios without any supplement (blue curve), supplemented with cholesteryl-hemisuccinate (green curve), with KCl (black curve) or both (red curve). Elution of solubilized membrane proteins separated on a Superose 6 10/300 GL column were compared with those of molecular weight markers (GE Healthcare Life Science) separated in the same column to determine their approximate size. The Elution of markers were: Blue dextran 2000, 2000 kDa at void volume 8 ml (red asterisk), thyroglobulin 669 kDa at 12.5 ml, ferritin 440 kDa at 14.5 ml, aldolase 158 kDa at 16.3 ml, conalbumin 75 kDa at 17.3 ml, ovalbumin 44 kDa at 17.6 ml. The green asterisk indicates the elution volume of GFP

To identify conditions that improve protein quality of solubilized GFP tagged PfKch1^1−1094^ and PfKch2^fl^ channels we performed FSEC analysis on membranes solubilized in detergents in the presence or absence of 5 mM KCl, and/or 0.026% (w/v) CHS. As shown in Fig. 8, addition of CHS increased solubilization efficiency for both channels in all detergents and resulted in more monodisperse elution profiles and increased solubility. Presence of 5 mM KCl during solubilization did not increase monodispersity in the absence of CHS. The Superose 6 elution profiles of the two proteins show that while GFP-PfKch2^fl^ has a promising FSEC profile in several detergents that would make it suitable for cryo-EM, PfKch1^1−1094^-GFP shows a less monodisperse elution profile.

### Ni-affinity purification of GFP tagged PfKch1^1−1094^ and PfKch2^fl^

**Fig. 9:**
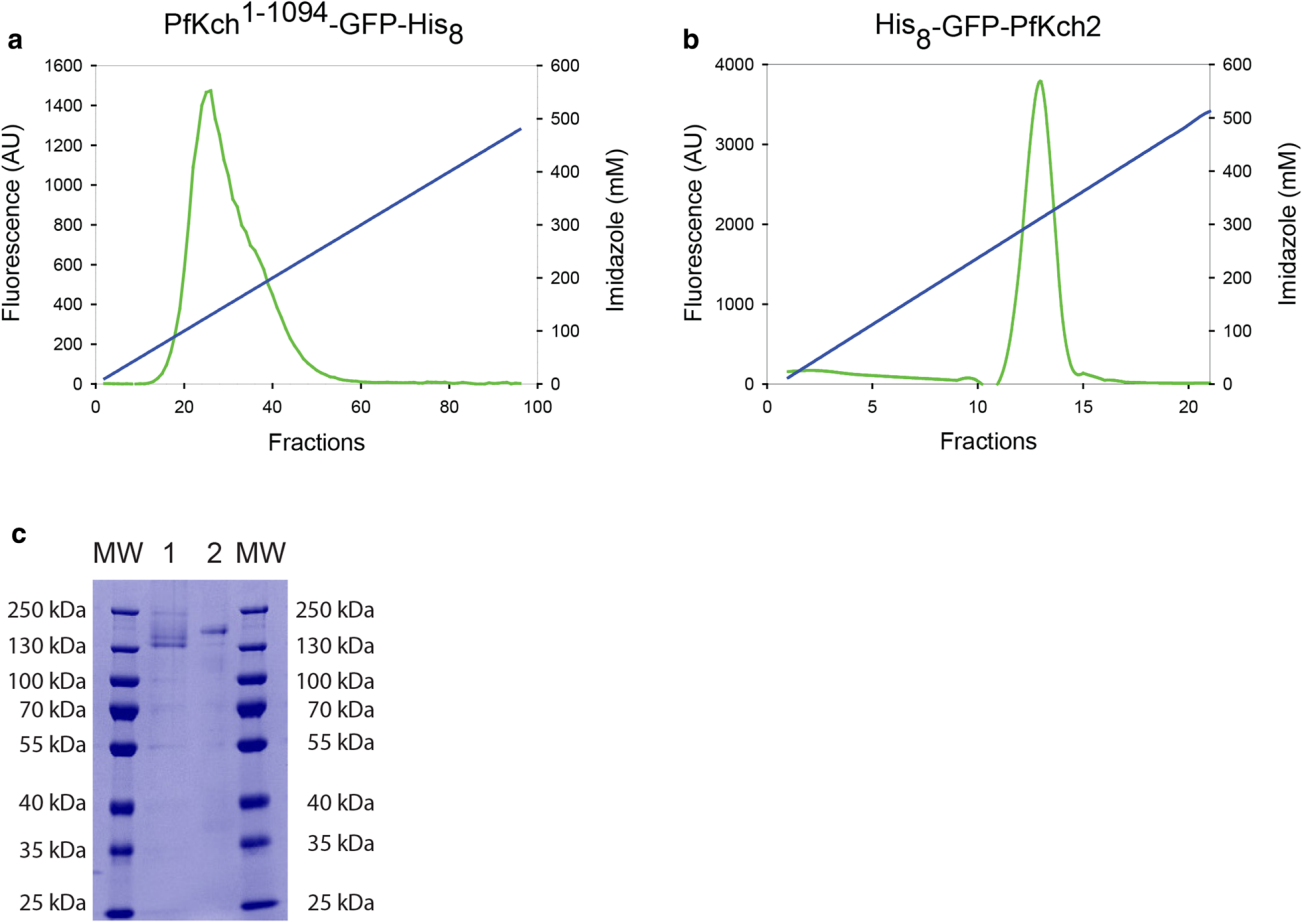
Purification by Ni-affinity chromatography of PfKch1^1−1094^-GFP and GFP-PfKch2^fl^. The two channels were solubilized in FOS-12 + CHS and loaded on HisTrap Ni columns over night at 4 °C. The proteins were eluted from the HisTrap columns using a linear imidazole gradient from 10-500 mM (blue lines). Fluorescence was measured in each fraction and used to generate the elution profiles (green curves). **a**: Purification of PfKch1^1−1094^-GFP and **b**: GFP-PfKch2^fl^. **c**: Coomassie Blue stain of the peak fractions from a and b separated in a 4–20% gradient Novex SDS-PAGE gel. Lanes 1: Mw marker 2:PfKch1^1-1094^-GFP 3: GFP-PfKch2^fl^ and 4: Mw marker

**Fig. 10:**
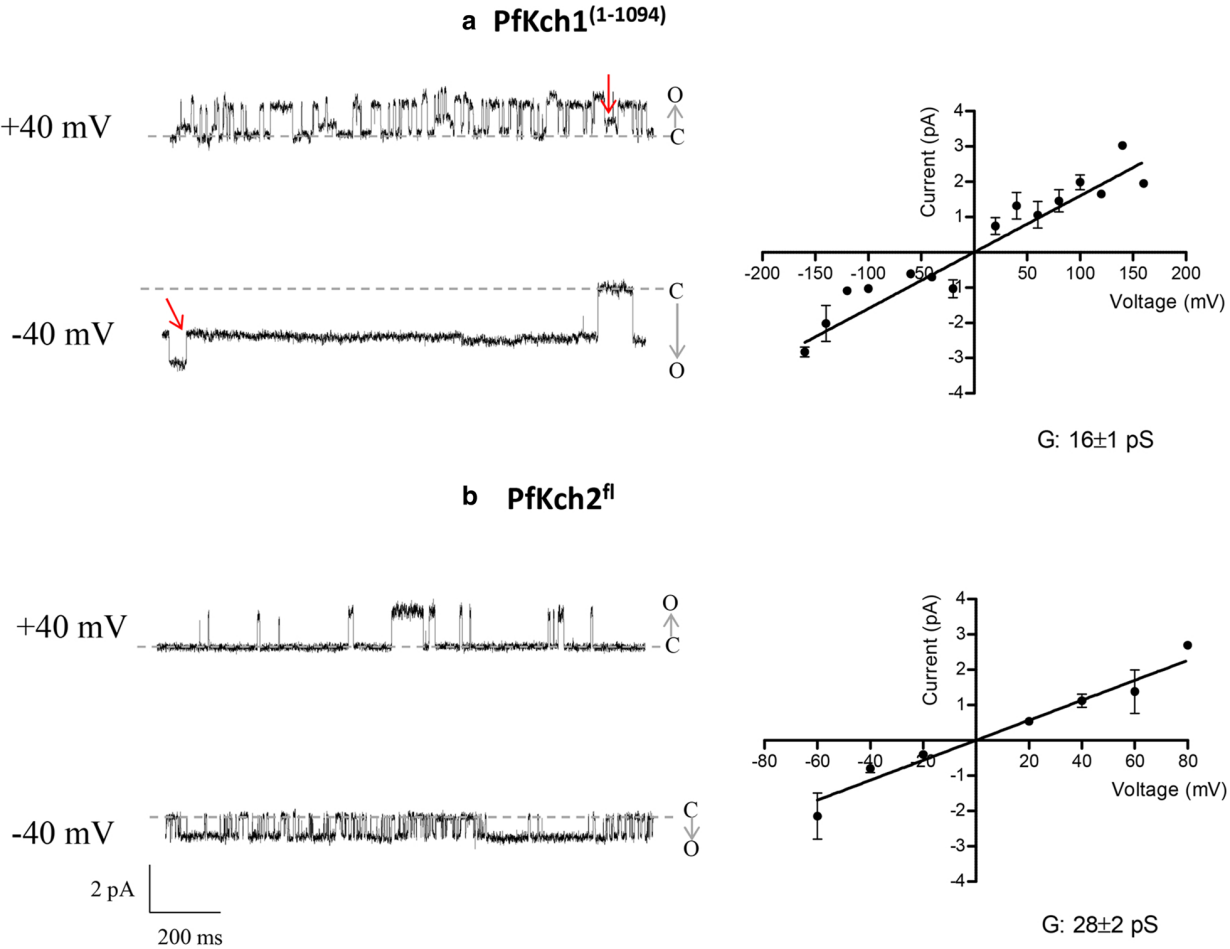
Functional reconstitution of purified PfKch1^1−1094^-GFP and GFP-PfKch2^fl^ into lipid bilayers. Solubilized and purified PfKch1^1−1094^-GFP and GFP-PfKch2^fl^ proteins were reconstituted into giant unilamellar vesicles (GUVs) consisting of 10 mM DPhPC and 1 mM cholesterol and measured in a planar lipid bilayer setup in symmetrical KCl solutions (135 mM) using the Nanion Port-a-Patch system. **a** The left panel shows the result of incorporation of a single PfKch1^1−1094^-GFP channel into the bilayer. Currents are recorded at −40 or +40 mV. Closed states of the channel (zero current) are indicated with a dashed line (**c**) and openings (o) are seen as upward and downward deflections at negative and positive potentials respectively. Sub-conductance states are indicated by red arrows. The right panel shows an IV-plot of single channel events recorded at different voltages ranging from −160 mV to +160 mV. The data are obtained from 7 independent experiments with one or more incorporated channels. b: Single channel currents of GFP-PfKch2^fl^ were recorded at -40 and +40 mV as described above. The left panel shows the results of incorporation of a single channel and open and closed states are indicated as in a. The right panel shows the IV-plot of single channel events recorded in 6 independent experiments.

Ni-affinity chromatography of FOS-12-CHS solubilized membranes revealed that PfKch1^1−1094^-GFP eluted as a relatively broad peak after the gradient reached 100 mM imidazole (Fig. 9a), whereas GFP-PfKch2^fl^ eluted as a more distinct peak, also at 100 mM imidazole (Fig. 10).

### PfKch1 and PfKch2 are predicted to encompass gating ring-forming RCK domains

To reveal the function of the water soluble parts of the PfKch1^fl^ and PfKch2^fl^ K^+^ channels, we performed a bioinformatics analysis using the Phyre^2^ software [32]. The search revealed that the 1195 amino acids C-terminal of PfKch1^fl^ show homology to regulator of conductance of K^+^ domains (RCK) that are known to form gating rings in K^+^ channels [33, 34]. The Phyre^2^ search produced a set of 3D models of the C-terminal of PfKch1^fl^ based on alignments to RCK gating structures from eukaryotic, archeon and prokaryotic K^+^ channels. The four highest scores were estimated to be homologous to the C-terminal of PfKch1^fl^ with 100% confidence. The hits included structures of the gating rings of large-conductance Ca^2+^-regulated K^+^ channels (SLO1 or BK channel,) from humans [33, 34] and zebrafish [35], and to the gating ring of the human SLO3 K^+^ channel [36]. The human BK channel gating ring yielded the highest scoring template, and a stretch of PfKch1^fl^ spanning residues 804 to 1964 was aligned to 601 residues of the BK channel comprising the RCK1 and RCK2 domains. The results suggest that the C-terminal part of PfKch1 contains an RCK1 domain from residues 810 to 1337 (Fig. 1b), however with an additional insertion of 92 residues in the loop between βD (green box I) and αD (green box II), and 177 residues in the loop between αG (green box II) and αH (green box III) (see detailed explanation of domain structure in extended data Fig. 1 in [37]). The alignment further suggested an RCK2 domain from residues 1495 to 1962 (Fig. 1a) is interrupted by an insertion of 122 amino acids in the loop between αQ (red box IV) and βO (red box V). An RCK1-RCK2 linker of 158 residues separates the two domains.

A Phyre^2^ analysis of PfKch2^fl^ also revealed homologies to RCK domains in the C-terminal 1097 amino acids as found in the above K^+^ channels of eukaryotic, archeon and prokaryotic origin. As with PfKch1^fl^, the human BK channel gating ring yielded the highest scoring template, and a stretch of PfKch2^fl^ spanning residues 421 to 1461 was aligned to 579 residues of the BK channel comprising the RCK1 and RCK2 domains. The results suggest that the C-terminal of PfKch2^fl^ contains an RCK1 domain from residues 421 to 715 (Fig. 1b, green box I), an RCK2 domain from residues 1166 to 1461 (Fig. 1b, red box II), and a 451 residues RCK1-RCK2 linker between the two domains (Fig. 1b). The presence of RCK domains in the *P. falciparum* K^+^ channels was further supported by comparison of the proposed RCK1 domains to the conserved sequence motifs in the family of RCK domains pointed out by Jiang et al. [38]. The conserved RCK family residues were also present in the *P. falciparum* K^+^ channels verifying the existence of the RCK domains. (Additional file 1: Figs. S2 and S3 show the detailed Phyre^2^ predictions.)

Transmembrane helices in the two K^+^ channels were predicted using the TOPCONS algorithm [39] and TMHMM [40]. As illustrated (Fig. 1b and d) the channels have a voltage-sensor domain (VSD) comprising helices S3-S6 and the pore-forming unit S7-S8 characteristic of K^+^ channels.

In conclusion, the results above suggest that both PfKch1^fl^ and PfKch2^fl^ may contain RCK1 and RCK2 domains in their C-terminals that form gating rings regulating channel gating in response to sensing of intracellular Ca^2+^ or other chemical stimuli. In addition, a number of transmembrane helices predicted in the N-terminals of the two channels suggest a rather complex structure.

### Purified PfKch1^1−1094^-GFP and GFP-PfKch2^fl^ proteins form functional channels in lipid bilayers

The complementation studies performed in the CY162 yeast strain (Fig. 3) show that the *Plasmodium* K^+^ channels are functional after expression in yeast. In order to prove integrity of the purified proteins and to assess their functionality after reconstitution, the purified PfKch1^1−1094^-GFP and GFP-PfKch2^fl^ proteins were reconstituted into giant unilamellar vesicles (GUVs). Planar lipid bilayers were generated from the GUVs on borosilicate glass chips with a resistance of 2–5 MΩ using the Nanion Port-a-Patch system (see Materials and Methods). All measurements were done in symmetrical 130 mM KCl solutions and in presence of 10 μM CaCl_2_. In the lipid bilayers, the reconstituted PfKch1^1−1094^-GFP showed clear single channel behavior, and openings and closings could be readily detected (Fig. 10a, left panel). The average single channel conductance was 16 ± 1 pS (Fig. 10a, right panel). A closer inspection of the single channel traces, reveal a number of apparent sub-conductance states, which are indicated by arrows (Fig. 10a).

In similar experiments, purified GFP-PfKch2^fl^ protein was reconstituted into planar lipid bilayers. In this case, clear single channel events could be detected as well (Fig. 10b, left panel), and the single channel conductance was estimated to 28 ± 2 pS (Fig. 10b, right panel). The reconstituted GFP-PfKch2^fl^ channels also occasionally showed behavior, which could reflect sub-conductance states (*data not shown*).

## Discussion

The increasing spread of resistance towards conventional malaria treatment makes the search for new drug-targets and medications ever more relevant. Traditional medicines such as chloroquine and sulfadoxine-pyrimethamine based remedies are aimed at the parasites hemoglobin metabolism [41] and folic acid synthesis [42], respectively. The mode of action of artemisinine based treatments is not yet fully understood [43, 44] and remains a controversial subject. It has previously been suggested that PfATP6, the *Plasmodium falciparum* version of sarcoplasmic-endoplasmic Ca^2+^ ATPase (SERCA) was the main target of artemisinin [45], but this has since been questioned [46, 47]. Other recent reports argue that the effect is haeme dependent and acts via a promiscuous binding mechanism, involving several targets [48].

Proteins involved in drug resistance mechanisms have been known for quite some time. The proteins are mainly localized intracellularly in the parasite and have been the subject of much scrutiny [49–52], in part to observe how resistance develops and to observe and continually monitor the spreading of resistance on a global scale. However, for the last two decades the membrane permeation pathways of the parasite have attracted increased attention [53–57]. The hope is to elucidate new ways of treating the disease through understanding and interfering with the parasite’s nutrient uptake and mechanisms of cell-homoeostasis regulation. Parasite homeostasis is particularly challenged when it travels from such diverse environments as the mosquito gut to the human bloodstream and the inside of erythrocytes. In this aspect it is noteworthy that *Plasmodium* parasites have the ability to alter the Na^+^/K^+^ ratio in the erythrocyte host, resulting in a reduced intracellular K^+^ level [58, 59]. It can be argued that, although quite different from each other, the overall variation of environments experienced by *Plasmodium* species is less than what free living eukaryotic organisms normally encounter, as suggested by Bushell et al. [6]. This in turn, they argue, could explain the lack of gene redundancy found in a *P. berghei* phenotypic screen, which inferred that, for normal growth in a single life cycle stage of the parasite, almost two-thirds of its genome were required [6]. For the intra-erythrocytic stage specifically however, this screen did not reveal the murine homologues of PfKch1 and PfKch2, to be essential.

In this study we have focused on the two K^+^ channels from the *P. falciparum* parasite, PfKch1 and PfKch2. In our hands these channels have resisted expression in mammalian cell lines and *Xenopus laevis* oocytes (*unpublished results*), and this led us to consider yeast expression. An interesting similarity between *Plasmodium* parasites and yeast is the very negative membrane potentials of approx. -95 mV found in parasite cells [60] and approximately -180 mV in yeast cells [61]. Both organisms generate their membrane potential through the action of a proton pump [61, 62], which creates an hyperpolarized membrane which may drive K^+^ uptake. This is contrary to the efflux of K^+^ seen in mammalian cells and *Xenopus laevis* oocytes [30]. As described in the present paper, the PfKch1 and PfKch2 channels can succesfully be expressed in a functional form in *Saccharomyces cerevisiae.* The process of quantifying and purifying the channel proteins has here been achieved through tagging with a GFP-His_8_ tail. This also served as a tool to determine the subcellular localization of the channels in yeast. We found that PfKchn1^fl^-GFP localized mainly in intracellular membranes while PfKch1^1−1094^ C-terminally tagged with GFP localized to the plasma membrane and PfKch2^fl^ N-terminally tagged with GFP accumulated in intracellular structures (Fig. 4). We did not investigate the reason for the observed difference in location between PfKchn1^fl^ and PfKchn1^1−1094^,but the most straightforward explanation is that parts of the most C-terminal 872 amino acids seem to compromise further transport from intracellular membranes such as the endoplasmic reticulum or Golgi to the plasma membrane. If this is related to the heterologous host or reflects a regulatory mechanism in the parasite controlling plasma membrane localization through interactions between the C-terminal of PfKch1 and unknown proteins may be worth addressing in future studies. In *P.* *falciparum*-infected human red blood cells (RBCs), PfKch1 has been reported to be expressed and transported to the plasma membrane of the RBC, while PfKch2 apparently was localized in intracellular compartments, especially in the late-stage merozoites [15]. Regarding PfKch1, however, this localization is difficult to recognize, as none of the many published functional studies on malaria-induced transport properties of the RBC plasma membrane has been able to demonstrate a new K^+^ permeability induced by the parasite [53–57]. The exact function and localization of these channels in vivo may still be debated. Our results support the notion that PfKch1, like the PbKch1, is the more important channel for blood stage parasite plasma membrane K^+^ transport as proposed by Ellekvist et al. [18] as the complementation observed in our studies was much more profound for this channel, than for PfKch2. However, the role of PfKch2 may be revealed in the future, quite possibly at an intra-parasitic level, which our localization observations seem to indicate.

Purified PfKch1^1−1094^-GFP and GFP-PfKch2^fl^ proteins could be reconstituted in lipid bilayers and both proteins show ion channel activity. The single channel traces shown in Fig. 10 represents the first measurements ever of the cloned *Plasmodium* encoded K^+^ channels. In addition, the measurements prove that the very large K^+^ channel proteins can be expressed in yeast, solubilized, purified and reconstituted with preservation of ion channel activity. Thus, our system constitutes a promising platform for future extensive structural and functional studies of these novel channels.

The single channel measurements allow us tentative insight into the single channel conductances for the *Plasmodium* K^+^ channels; which we find to be 16 pS for PfKch1^1−1094^-GFP and 28 pS for GFP-PfKch2^fl^ (Fig. 10, right panels). Inspection of the single channel traces in Fig. 10 (left panels) suggests an apparent voltage-sensitivity. For both channels, the open probability is higher at negative voltages as compared to positive voltages. A voltage dependent gating of the channels is indeed consistent with our bioinformatics analysis, which shows the presence of three and two charged amino acids in the S4 segment of PfKch1 and PfKch2, respectively. However, it is difficult to draw final conclusions concerning voltage regulation based on the present study, since the direction of the incorporated ion channels in the lipid bilayers is not known. Also, we have indications that the *Plasmodium* channels could be regulated by Ca^2+^. Initial attempts at incorporation of the reconstituted channels into lipid bilayers showed that the presence of 10 μM Ca^2+^ greatly increased the success rate. Given that we have identified RCK domains in both channel proteins, it seems tempting to suggest that the channels could be regulated by Ca^2+^.

The accumulation of the *P. falciparum* K^+^ channel proteins produced under optimal conditions in a bioreactor amounted to 1–1.6% of total membrane protein content, which is astoundingly high considering the size and complexity of the proteins. This is a comfortable starting point to explore cryo-EM structure determinations, considering that similar studies of large and complex recombinant membrane proteins such as the GPCR receptors, succeeded with an expression level of only 0.2% expressed receptor protein per total membrane protein content [63].

Choosing the right detergent for efficient solubilization of membrane proteins is a considerable bottleneck in structure determinations [64]. Our detergent screen showed that we can solubilize and extract reasonable amounts of functional channels from crude yeast membranes. The efficiency of the Ni-affinity purification of PfKch1^1−1094^-GFP and GFP-PfKch2^fl^ was revealed by the purity of samples as seen on the Coomassie stain of the SDS-PAGE separated fractions.

## Conclusion

The overall conclusion of this study shows that it is possible to express and purify the large and complex, membrane spanning PfKch1^1−1094^-GFP and GFP-PfKch2^fl^ proteins in functional forms from yeast. It is our hope that the described heterologous expression platform can contribute to recombinant production of the notoriously difficult to produce *P. falciparum* proteins. We have previously obtained similar results with the human hERG K^+^ channel using the same approach [27]. Together, these results establish a cost-effective procedure that may also be useful for characterization of other eukaryotic difficult-to-express ion channels. We have presented the first electrophysiological data of two P. *falciparum* K + channels. These pioneering results pave the way for structural studies and a more comprehensive electrophysiological characterization of the *P. falciparum* K^+^ channels that potentially may turn out to be important drug targets, and may reveal more insights into the life cycle of the malaria parasite.

## Methods

### Yeast strains

*S. cerevisiae* strains CY162 (*MATα trk1Δ trk2Δ::HIS3 ura3*-*52 his4*-*15, his3Δ200*) [30], PAP7111 (CY162 carrying a *PMA1*::mcherry fusion) was used for the complementation assay and PAP1500 (α *ura3*-*52 trp::GAL10*-*GAL4 lys2*-*801 leu2Δ1 his3Δ200 pep4::HIS3 prb1Δ1.6R can1 GAL*) [65] was used as host for production of PfKch1^1−1094^-GFP and GFP-PfKch2^fl^ channels for purification.

### Recombinant plasmid construction

Full-length or fragments of PfKch1 or PfKch2 codon optimized for expression in *Xenopus* oocytes or *Saccharomyces cerevisiae* were purchased from Geneart, DE, and GenScript, USA, respectively. All yeast expression plasmids were generated by in vivo homologous recombination in *S. cerevisiae* between *Bam*HI, *Hind*III digested pEMBLyex4 [66] and PCR fragments encoding full length or parts of PfKch1 or PfKch2 cDNA. In-frame C- or N-terminal tagging of PfKch1^1−1094^, PfKch1^fl^ or PfKch2^fl^ with yEGFPs [67] were constructed by in vivo recombination between *Bam*HI, *Hind*III digested pEMBLyex4 expression vector and PfKch1 or PfKch2 PCR fragments and a GFP PCR fragment amplified with primers adding either N-terminal or C-terminal TEV sites and HIS_8_ tags. Homologous recombination was achieved by transforming CY162 [30] or PAP1500 [65] according to the method of Gietz and Schiestl [68]. Correct sequences of tagged constructs were verified by sequencing services offered at Eurofins Genomics, Germany, on purified plasmids.

### Functional complementation in liquid media

Transformed CY162 yeast cells were grown at 25 °C in liquid synthetic minimal medium containing 2% glucose as sole carbon source and 100 mM KCl. Exponentially growing cells were harvested, washed four times in sterile 18 mΩ H_2_O and subsequently re-suspended in 18 mΩ H_2_O to an OD_450_ = 0.5 and used to inoculate the minimal media containing wells in the growth assay to an OD_450_ = 0.05. Complementation assays were performed in 96-well plates at a volume of 200 ml. All growth experiments were performed in amino acid supplemented minimal medium containing 2% glucose, 2% galactose and KCl concentrations from 0 mM til 100 mM. The medium was buffered with 100 mM TES/Tris, pH = 6. Plates were incubated at room temperature and OD_450_ was measured at least twice a day in a plate-reader (Multiskan RC, Thermo Labsystems).

### Protein production

Cultures were set up by inoculating 5 ml of leucine supplemented synthetic minimal (SD) medium and incubated O/N at RT. When saturated, 100 μl were transferred to media with no leucine, to select for high plasmid copy numbers. These cultures were subsequently transferred to 1 l Expression media (SD medium with valine (150 µg/L), tyrosine (30 mg/L), tryptophan (20 mg/L), threonine (200 mg/L), serine (375 mg/L), proline (20 mg/L), phenylalanine (50 mg/L), lysine (30 mg/L), histidine (20 mg/L), glutamic acid (100 mg/L), cysteine (20 mg/L), aspartic acid (100 mg/L), arginine (20 mg/L), alanine (20 mg/L), glycerol (3% v/v) and glucose (0.5% w/v)) to an OD_450_ of 0.05. Cultures were incubated at room temperature. At OD_450_ = 1, each culture was separated in two that were temperature equilibrated at 15 °C or 30 °C, and then induced to express the recombinant channels by addition of induction medium (expression medium with 20% galactose instead of 0.5% glucose) at a final concentration of 2% galactose. Cultures were harvested after 12, 24, 48, 72 and 96 h of incubation.

### Bioimaging of live cells

The localization of GFP tagged channels in yeast was determined by bioimaging of live cells that had been induced for channel production for 24–48 h. Images were taken using an Optronics Magnafire model S99802 camera coupled to a Nikon Eclipse E600 microscope at a 1000x magnification.

### Preparation of crude membrane fractions

Crude yeast membranes were prepared by homogenizing cells with glass beads as described previously [69]. In short, the resuspension of the 1 l cell culture pellets were carried out in 10 ml ice cold lysis buffer with a pH of 7.5 (10% glycerol (v/v), 1 mM EGTA, 1 mM EDTA and 25 mM imidazole) with protease inhibitors (Aprotinin (1 μg/ml), Benzamidine (1 mM), Chymostatin (1 μg/ml), Leupeptin (1 μg/ml), Pepstatin (1 μg/ml) and PMSF (1 mM)). Cell suspensions were vortexed 5 x 1 min and kept on ice between vortexing cycles. The supernatants were collected and the remaining beads washed with lysis buffer several times, to generate samples of 50 ml total volume. A 10 min centrifugation at 3000 rpm and 4 °C, were carried out in an SS-34 Sorvall rotor, to pellet cell debris in the samples. The crude membrane fractions were then isolated by a 1.5 h ultra-centrifugation at 40,000 rpm and 4 °C using a Sorvall 70TI rotor. The pellets containing the crude membranes were then re-suspended in 3 ml lysis buffer containing protease inhibitors (as described above) and homogenized manually with a Potter–Elvehjem homogenizer and kept at −80 °C.

### Protein and GFP quantification

A standard BCA assay [70] was used to determine the total membrane protein content of crude yeast membrane fractions. A kit (Sigma, USA) was used according to the manufacturer´s specifications, applying known concentrations of chicken ovalbumin for the standard curve. The fluorescence content was quantified by resuspending 25 μg crude membrane proteins in a total of 200 μl buffer (10 mM imidazole, 10% glycerol, 200 mM NaCl and 20 mM phosphate) at pH 7.0 in 96 well white microplates (Nucleon Nunc) and measuring their GFP fluorescence. Measurements were done in a Fluoroskan Ascent spectro fluorometer (Thermo Scientific), using 485 nm excitation and 520 nm emision filters. From a previously generated standard curve of purified GFP mixed with yeast membranes [25, 26] fluorescence could be converted to pmol K^+^ channel-GFP.

### Solubilization and detergent screen

Solubilization of crude membranes, from cells grown at 15 °C was done by incubation in buffer A (10% glycerol, 0.5 M NaCl, 10 mM imidazole, 25 mM Tris–HCl, 0.5 mM EDTA and 0.5 mM EGTA pH 7.6) supplemented with protease inhibitors (Aprotinin (1 μg/ml), Benzamidine (1 mM), Chymostatin (1 μg/ml), Leupeptin (1 μg/ml), Pepstatin (1 μg/ml)and PMSF (1 mM)) at protein:detergent:CHS ratios (w/w) of 1:2:0.7; 1:3:1 or 1:4:1.4. The screen included detergents LDAO, Lauryldimethylamine Noxide; FOS-12, n-dodecylphosphocholine; DDM, n-dodecyl-β-d-maltopyranoside;Cymal-5, 5-Cyclohexyl-1-pentyl-β-d-maltoside; C_12_E_8_, Octaethylene glycol monododecyl ether; DM, n-decyl-β-d-maltopyranoside; CHAPS, 3-[(3chol-amidopropyl)- dimethylammonio]-1-propane sulfonate/N,N-dimethyl-3-sulfo-N-[3-[[3a,5b,7a,12a)-3,7,12- tri–hydroxy-24-oxocholan-24- yl]amino] propyl]-1-propana-miniumhy-droxide and Octyl glucoside. Detergents were from either Affymetrix, UK and of Anagrade quality or from GLYCON Biochemicals, Germany. Solubilization was carried out under slow rotation at 4 °C for 1 h. Solubilized proteins were collected after pelleting cell debris by 30 min of ultra-centrifugation 70,000 rpm and 4 °C using a Beckman Optima™TLX ultracentrifuge fitted with an S.N. 96U 826 rotor. Fluorescence estimation was done as before in 96 well white microplates, using the spectro fluorometer (Fluoroskan Ascent, Thermo Scientific) with buffer as a blank. Excitation was at 485 nm and emission at 520 nm. The percentage of fluorescence of solubilized sample supernatant, as compared to fluorescence in the initial crude membrane fractions, was taken as a measure of solubilization efficiency.

### FSEC

Fluorescence detection size exclusion chromatography (FSEC) analysis of solubilized crude membranes was done on an ÄKTA Purifier (GE Healthcare, USA) using a Superose 6 10/300 column and FSEC buffer (20 mM TRIS–HCl, 0.15 M NaCl, 0.03% DDM pH 7.5). The samples were analyzed with and without addition of CHS 0.026% (w/v) and/or 5 mM KCl. The Superose 6 10/300 column effluent was coupled to a fluorescence detector (Shimadzu Prominence RF-20A). This facilitated fluorescence measurements and visualization of the GFP protein elution profiles. Molecular weight estimation of the solubilized channels was done by a comparison to the HMW calibration kit from GE Healthcare dissolved in FSEC buffer to a concentration of 20 mg/ml. The molecular masses of the kit components were: Blue Dextran 2000 kDa–the elution volume of which was used as definition of void volume; Thyroglobulin 669 kDA; Ferritin 440 kDa; Aldolase 158 kDa; Conalbumin 75 kDa; Ovalbumin 43 kDa.

### Ni–NTA affinity purification

To purify the PfKch1^1−1094^-GFP channel, they were first solubilized in buffer A at a protein:FOS-12:CHS ratio of 1:3:1 (w/w/w) under slow rotation for 2 h at 4 °C and subsequently centrifuged in a Beckman Optima™TL200 ultracentrifuge for 30 min at 70,000 rpm and 4 °C, to get rid of any unsolubilized material. Supernatants containing solubilized membrane proteins were collected and diluted in Buffer A with protease inhibitors, but without EDTA or EGTA, to reach a detergent concentration of 0.75 mg/ml corresponding to 1.5 times CMC for FOS-12 and a CHS concentration of 0.26 mg/ml. The diluted samples were setup to bind to a 5 ml HisTrap™HP (GE healthcare Life science) column, by running it over the column in a loop O/N at a pace of 1 ml/min at 4 °C. The samples were then separated by a 10-500 mM linear imidazole gradient on the ÄKTA Purifier (GE Healthcare, USA) by a 1 ml/min flow. A total of 95 1 ml fractions were collected, and fluorescence in each fraction was determined using a spectro fluorometer (Fluoroskan Ascent, Thermo Scientific, USA) with buffer used to estimate background fluorescence. Excitation was at 485 nm and emission at 520 nm. The GFP-PfKch2 channels were solubilized at a protein:FOS-12:CHS ratio of 1:1:0.3. The procedure was the same as for PfKch1^1−1094^-GFP channels but using a 1 ml HisTrap™HP (GE healthcare Life science) column instead and washing and eluting at a pace of 0.4 ml/min increase in imidazole concentration from 10 mM to 500 mM. A total of 21 fractions of 1 ml each was collected and fluorescence measured as described above. Purified fluorescence peak fractions of PfKch1^1−1094^-GFP and GFP-PfKch2 were subsequently visualized after SDS-PAGE separation in a 4–20% gradient Novex gel (Invitrogen) by Coomassie staining.

### Lipid bilayers and single channel recordings

Purified PfKch1^1−1094^-GFP and GFP-PfKch2^fl^ proteins were reconstituted into Giant Unilamellar Vesicles (GUVs) to assess their functional properties essentially as described earlier [71]. GUVs were prepared by an electro formation method using Vesicle Prep Pro (Nanion Technologies, Germany) with 10 mM 1,2-diphyntanoyl-sn-glycero-3-phosphatidylcholine (DPhPC) (Avanti Polar Lipids, USA) and 1 mM cholesterol (Sigma), both of them dissolved in chloroform and using 1 M D-Sorbitol as intra-GUV solution. The average size of GUVs formed by this method ranged between 30 and 100 mm. To prepare proteoliposomes, purified GFP-PfKch2^fl^ protein with a concentration of 33 ng/ml or PfKch1^1−1094^-GFP with a concentration of 17 ng/ml was mixed with the GUV solution and incubated at room temperature for 20 min. Detergents were removed by addition of absorbent Bio-Beads™SM-2 (Bio-Rad) for 1 h at room temperature, followed by an overnight incubation at 4 °C. Bio-Beads™ were discarded after a short spin. The proteoliposome solution was either used right away or stored at 4 °C for up to 4 days prior to recordings. The formation of lipid bilayers, as well as the channel activity was monitored on a Port-a-Patch (Nanion Technologies, Germany). Lipid bilayers were formed on NPC-1 borosilicate glass chips with a resistance of 2–5 MΩ using symmetrical K^+^ concentrations on both sides of the bilayer (130 mM KCl, 10 mM HEPES, pH = 7). During experiments, Ca^2+^ was directly added to the upward facing side of the chip to a final concentration of 10 μM. Data were recorded at room temperature (22 °C) at a sampling rate of 50 kHz and filtered using a low-pass Bessel filter at 2 kHz with an EPC-9 HEKA amplifier (HEKA Elektronik, Germany). Single channel events were detected and analyzed in Clampfit 10 (Molecular Devices, USA).

## Supplementary information

**Additional file 1:** **Figure S1.** Topology analysis of PfKch1 and PfKch2. **Figure S2.** Phyre^2^ generated alignment between PfKch1 and *Aplysia* Slo1. **Figure S3.** Phyre^2^ generated alignment between PfKch2 and *Aplysia* Slo1.

## Data Availability

The datasets used and/or analysed during the current study are available from the corresponding author on reasonable request.
