# Supplementary material for: Purification and initial characterization of Plasmodium falciparum K+ channels, PfKch1 and PfKch2 produced in Saccharomyces cerevisiae
Source: Microb Cell Fact. 2020 Sep 21;19:183. doi: 10.1186/s12934-020-01437-7 (PMC7507820; doi:10.1186/s12934-020-01437-7)
Supplement: Supplementary file 1 — Additional file 1: Figure S1. Topology analysis of PfKch1 and PfKch2. Figure S2. Phyre2 generated alignment between PfKch1 and Aplysia Slo1. Figure S3. Phyre2 generated alignment between PfKch2 and Aplysia Slo1. [file 12934_2020_1437_MOESM1_ESM.docx]

**Additional File 1**

**Purification and initial characterization of *Plasmodium* *falciparum* K**^+^ **channels, PfKch1 and PfKch2 produced in *Saccharomyces cerevisiae***

**Karen Molbaek**^1,2^**, Maria Tejada**^2^**, Christina Hoeier Ricke**^2^**, Peter Scharff-Poulsen**^1^**, Peter Ellekvist**^3^**, Claus Helix-Nielsen**^4,5,6^**, Nirbhay Kumar**^7^**, Dan A. Klaerke**^2,*^**, and Per Amstrup Pedersen**^1,*^

^1^University of Copenhagen, Department of Veterinary and Clinical Animal Science, Frederiksberg, 1870, Denmark.

^2^University of Copenhagen, Department of Biology, Copenhagen, 2100, Denmark

^3^Herlev-Gentofte Hospital, Medical Department, Herlev, 2730, Denmark

^4^Aquaporin A/S, Kgs Lyngby, 2800, Denmark

^5^Technical University of Denmark, Department of Environmental Engineering, Kgs Lyngby, 2800, Denmark

^6^University of Maribor, Laboratory for Water Biophysics and Membrane Technology, Maribor, 2000, Slovenia

^7^Department of Global Health, Milken Institute School of Public Health, George Washington University Washington DC 20052-0066, United States of America

Correspondence: [*dk@sund.ku.dk](mailto:*dk@sund.ku.dk), [*papedersen@bio.ku.dk](mailto:*papedersen@bio.ku.dk)

**Contents**

Fig.S1: Topology analysis of PfKch1 and PfKch2

Fig.S2: Phyre2 generated alignment between PfKch1 and *Aplysia* Slo1

Fig.S3: Phyre2 generated alignment between PfKch2 and *Aplysia* Slo1

**Topology prediction of the N-terminal 569 amino acids from PfKch1 using the TOPCONS (1) software at (**[**http://topcons.cbr.su.se/**](http://topcons.cbr.su.se/)**) that predicts a topology model from the five algorithms shown below.**

|  |
| --- |


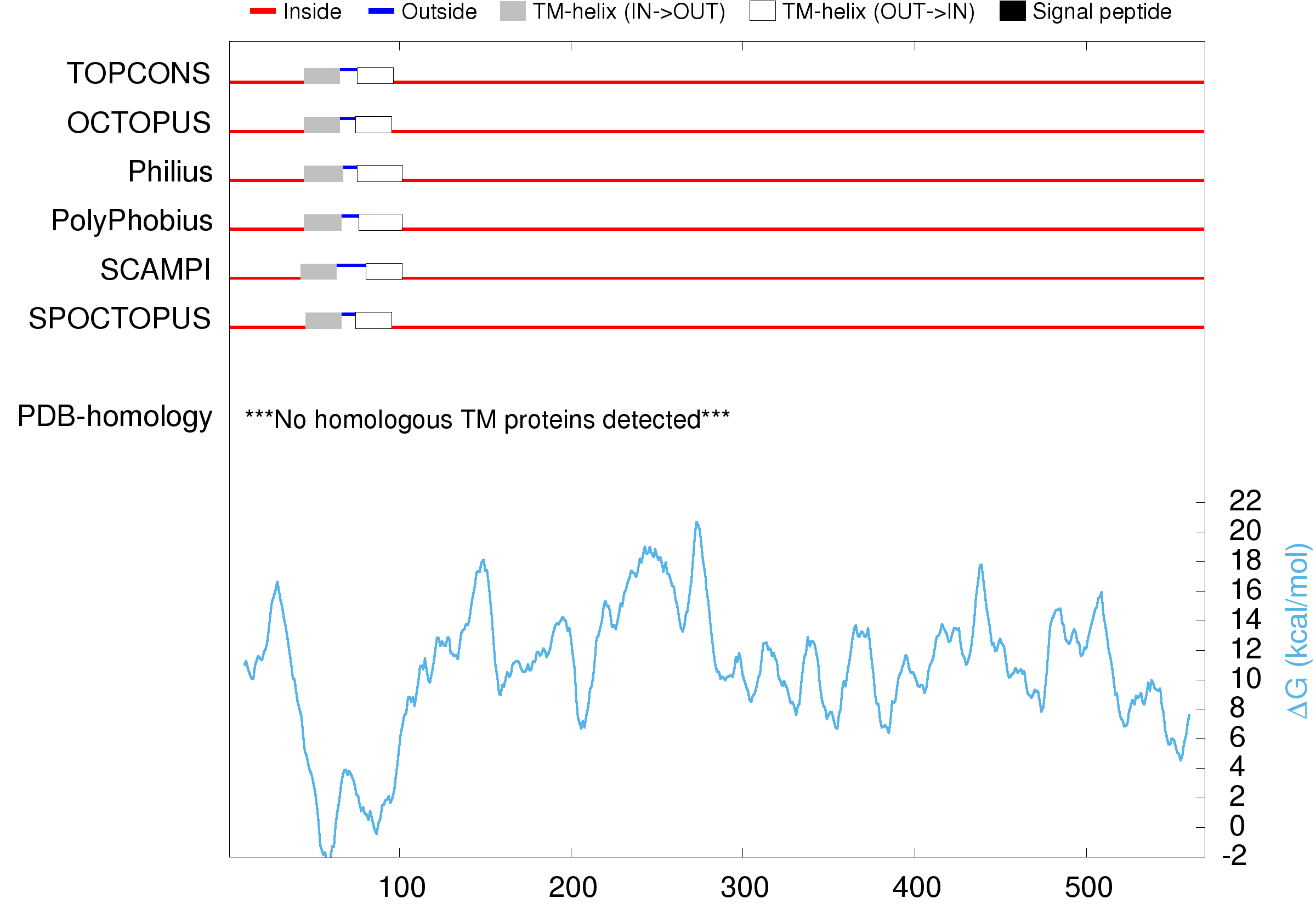


**A larger image of the TOPCONS analysis shown above**


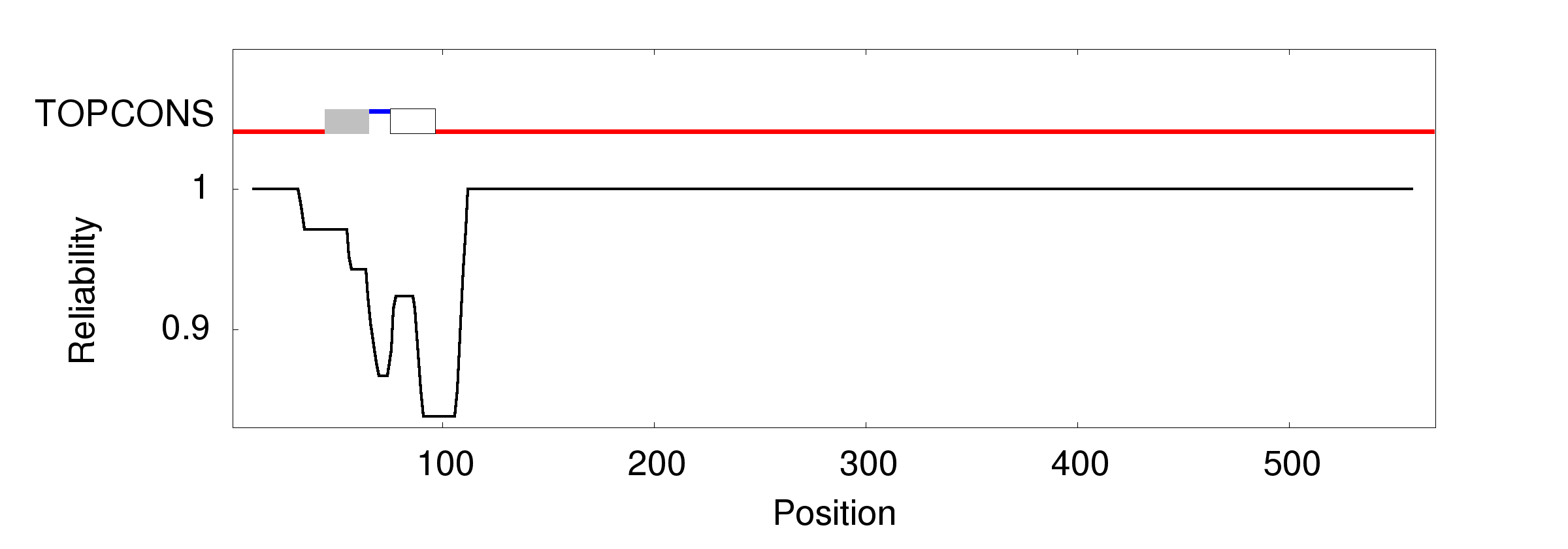


| **Predicted signal peptide and TM-helix positions (position starting from 1):**   \| TOPCONS \| **TM1:**45-65, \| **TM2:**76-96 \| \| --- \| --- \| --- \| \| OCTOPUS \| **TM1:**45-65, \| **TM2:**75-95 \| \| Philius \| **TM1:**45-67, \| **TM2:**76-101 \| \| PolyPhobius \| **TM1:**45-66, \| **TM2:**77-101 \| \| SCAMPI \| **TM1:**43-63, \| **TM2:**81-101 \| \| SPOCTOPUS \| **TM1:**46-66, \| **TM2:**75-95 \| \| PDB-homology \| ***No homologous TM proteins detected*** \| \| |
| --- | --- | --- | --- | --- | --- | --- | --- | --- | --- | --- | --- | --- | --- | --- | --- | --- | --- | --- | --- | --- | --- |

**TMHMM (2) (**<https://services.healthtech.dtu.dk/service.php?TMHMM-2.0>**)** **prediction of the transmembrane topology of the N-terminal 569 amino acids in PfKch1.**

# WEBSEQUENCE Length: 569

# WEBSEQUENCE Number of predicted TMHs: 2

# WEBSEQUENCE Exp number of AAs in TMHs: 46.6447100000001

# WEBSEQUENCE Exp number, first 60 AAs: 16.15112

# WEBSEQUENCE Total prob of N-in: 0.51865

# WEBSEQUENCE POSSIBLE N-term signal sequence

WEBSEQUENCE TMHMM2.0 inside 1 44

WEBSEQUENCE TMHMM2.0 TMhelix 45 67

WEBSEQUENCE TMHMM2.0 outside 68 76

WEBSEQUENCE TMHMM2.0 TMhelix 77 96

WEBSEQUENCE TMHMM2.0 inside 97 569


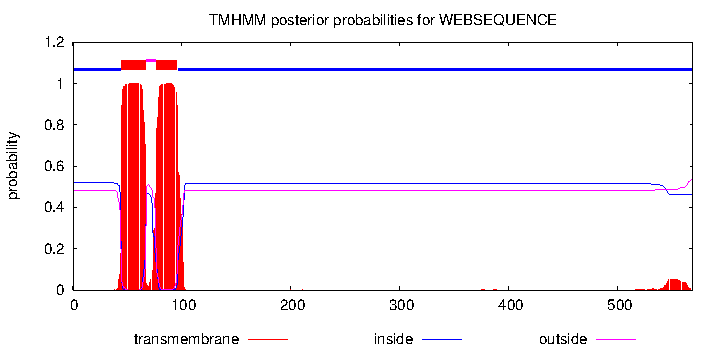


**Topology prediction of the N-terminal 139 amino acids of PfKch2 using the TOPCONS software (1) (**[**http://topcons.cbr.su.se/**](http://topcons.cbr.su.se/)**) that predicts a topology model from the five algorithms shown below.**

**
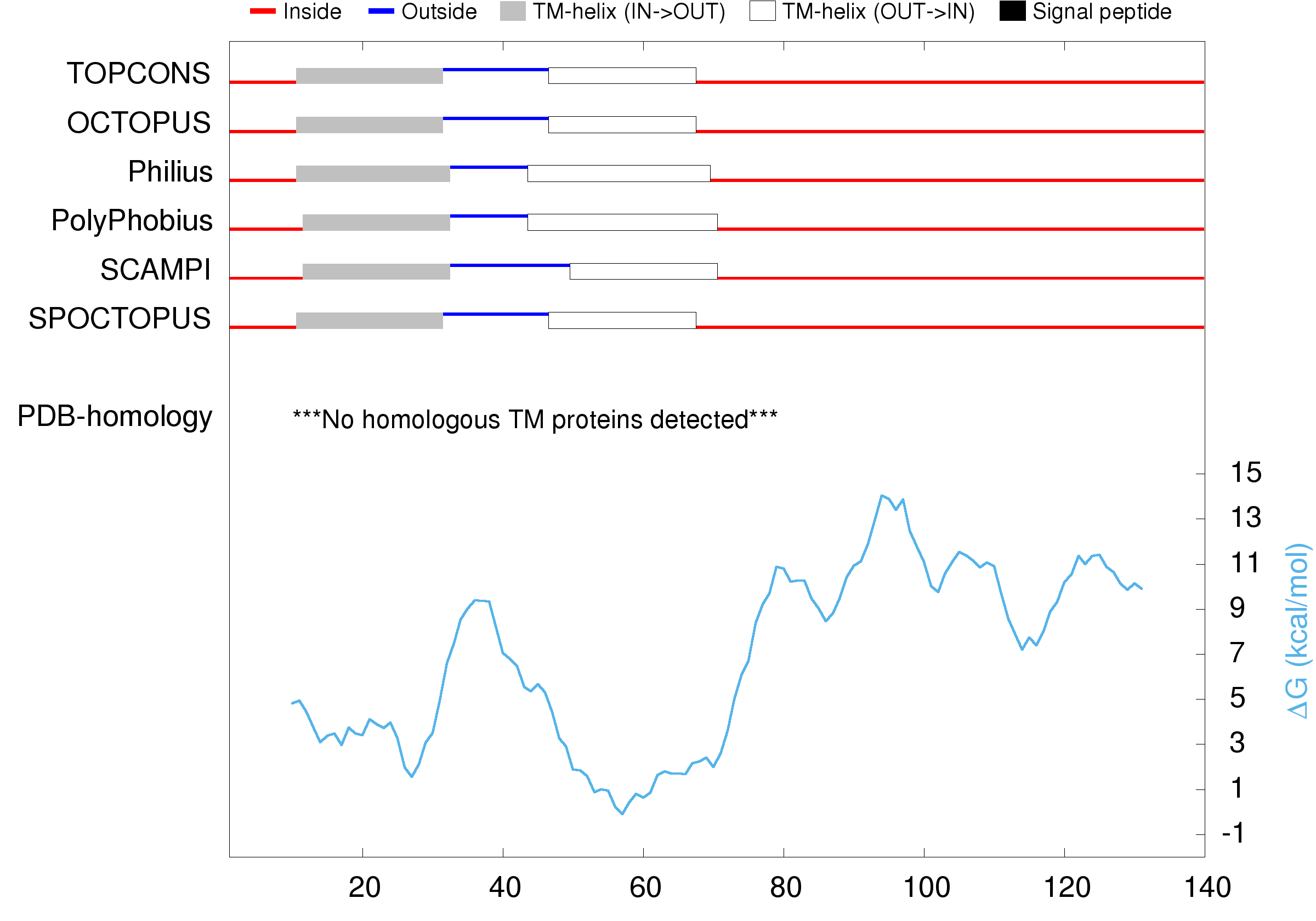
**

**A larger image of the TOPCONS results above**

**
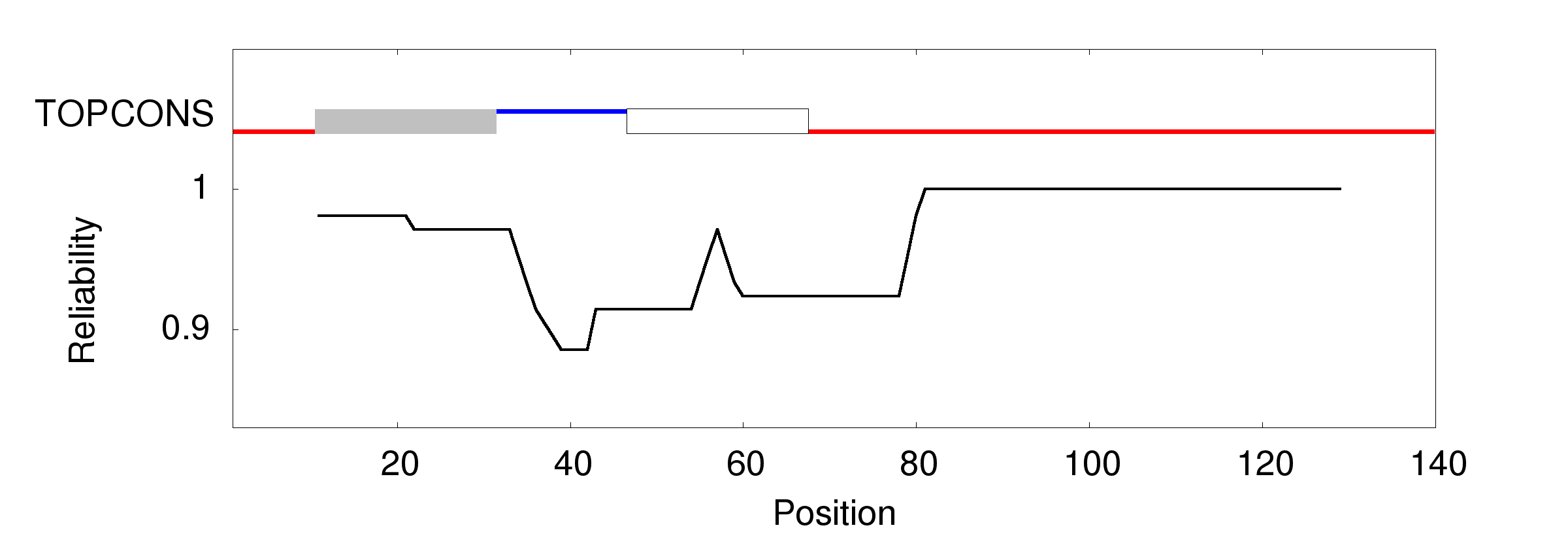
**

**TMHMM (2) (**<https://services.healthtech.dtu.dk/service.php?TMHMM-2.0>) **prediction of the transmembrane topology of the N-terminal 139 amino acids in PfKch2.**

# WEBSEQUENCE Length: 139

# WEBSEQUENCE Number of predicted TMHs: 2

# WEBSEQUENCE Exp number of AAs in TMHs: 46.33518

# WEBSEQUENCE Exp number, first 60 AAs: 36.59253

# WEBSEQUENCE Total prob of N-in: 0.97011

# WEBSEQUENCE POSSIBLE N-term signal sequence

WEBSEQUENCE TMHMM2.0 inside 1 11

WEBSEQUENCE TMHMM2.0 TMhelix 12 34

WEBSEQUENCE TMHMM2.0 outside 35 43

WEBSEQUENCE TMHMM2.0 TMhelix 44 66

WEBSEQUENCE TMHMM2.0 inside 67 139

| 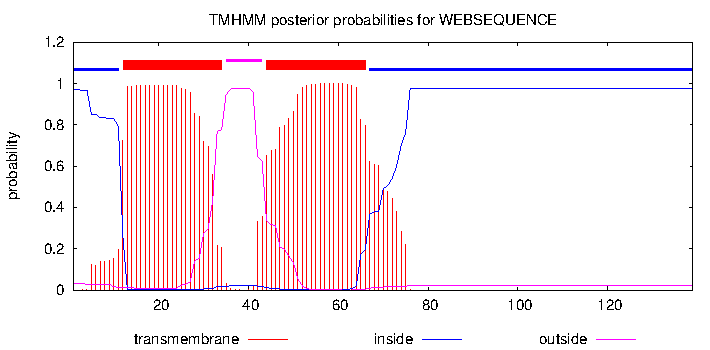 |  |  |
| --- | --- | --- |
|  |  | |

**Fig. S1:** Bioinformatics prediction of the transmembrane topology of the 569 and 139 N-terminal amino acids of PfKch1 and PfKch2 that could not be predicted by the Phyre^2^ software used to generate the homology models in Fig.1. The prediction was performed using the TOPCONS (1) and the TMHMM (2) algorithms.

**
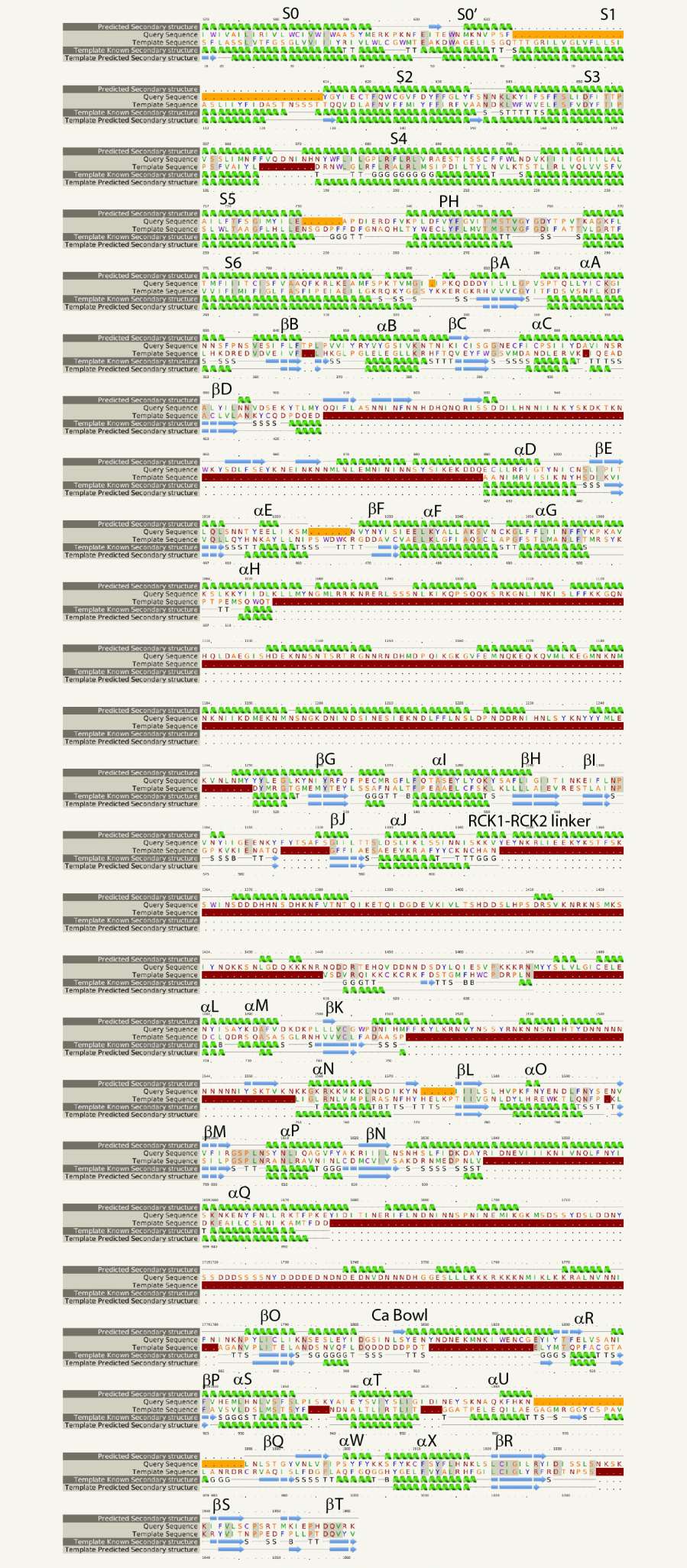
**

**Fig. S2 Sequence alignment of Pfkch1 and *Aplysia* Slo1.**

The amino acid sequences of Pfkch1 (Query Sequence) and *Aplysia* Slo1 (Template Sequence) were aligned using the Phyre^2^ alignment software (3). The secondary structure elements found in the *Aplysia* Slo1 structure (4) are indicated above the alignment. Pfkch1 residues 570 to 1961 were aligned to *Aplysia* Slo1 with 23 % sequence identities and a 100 % confidence that the two sequences are homologous.


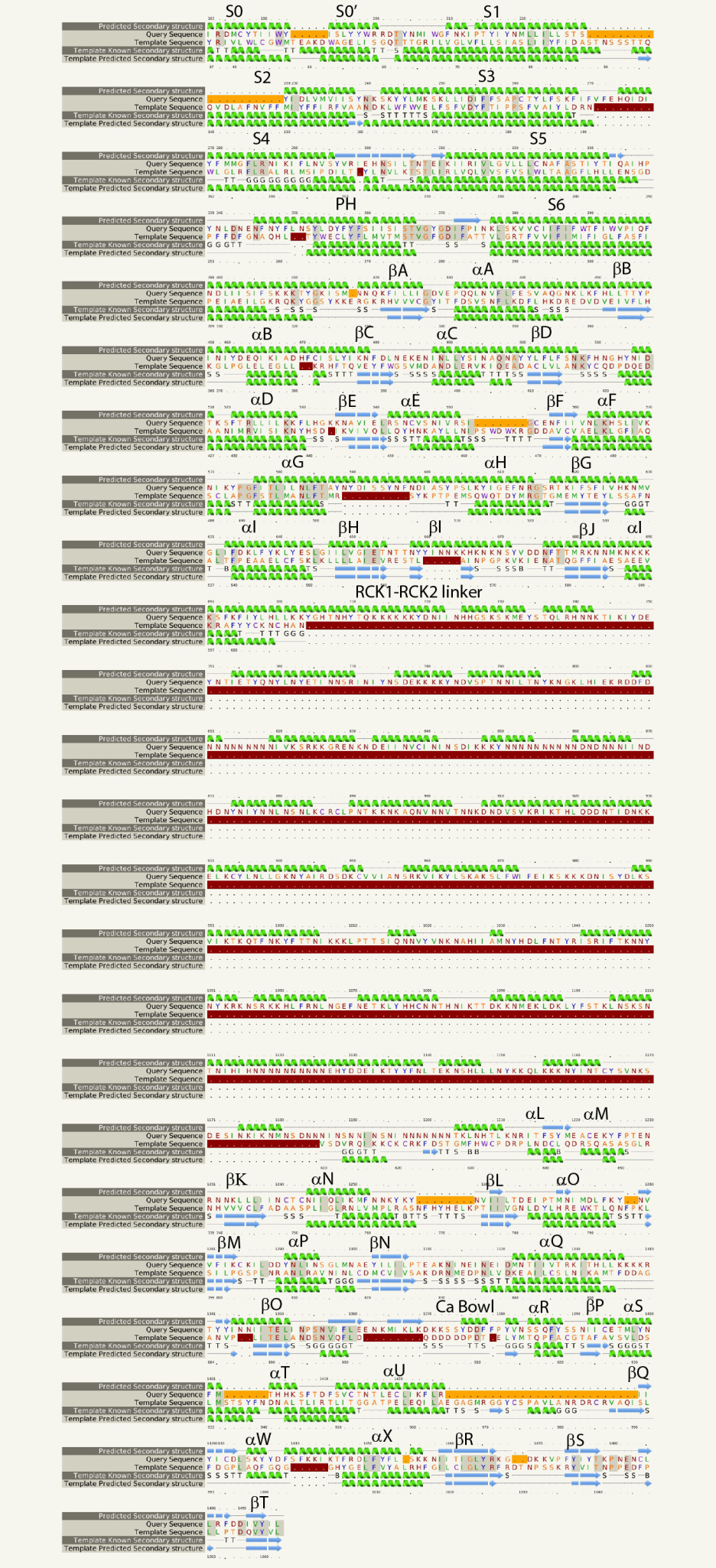


**Fig. S3 Sequence alignment of Pfkch2 and *Aplysia* Slo1.**

The amino acid sequences of Pfkch2 (Query Sequence) and *Aplysia* Slo1 (Template Sequence) were aligned using the Phyre^2^ alignment software (3). The secondary structure elements found in the *Aplysia* Slo1 structure (4) are indicated above the alignment. Pfkch2 residues 183 to 1495 were aligned to *Aplysia* Slo1 with 31 % sequence identities and a 100 % confidence that the two sequences are homologous.

**References**

1. Tsirigos, K.D., Peters,C., Shu, N., Käll, L., & Elofson, A. The TOPCONS web server for consensus prediction of membrane protein topology and signal peptides. Nucleic Acids Research. 2015 May 12: 43 (W1): W401-407; doi:10.1093/nar/gvk485.
2. Krogh, A., Larsson, B., von Heijne, G. & Sonnhammer, E. Predicting transmembrane protein topology with a hidden Markov model: Application to complete genomes. J Mol Biol. 2001 Jan 19;305(3):567-80; doi :10.1006/jmbi.2000.4315
3. Kelley, L. A., Mezulis, S., Yates, C. M., Wass, M. N. & Sternberg, M. J. E. The Phyre2 web portal for protein modeling, prediction and analysis. Nat Protoc. 2015 Jun;10(6):845-58; doi: 10.1038/nprot.2015.053.
4. Tao, X., Hite, R. K. & MacKinnon, R. Cryo-EM structure of the open high-conductance Ca^2+^-activated K+ channel. Nature. 2017 Jan 5;541(7635):46-51; doi: 10.1038/nature20608
